# Multifunctional and Hierarchical Porous ZIF‐8: Amine and Thiol Tagged via Mixed Multivariate Ligand Strategies for Enhanced CO_2_ and Iodine Adsorption

**DOI:** 10.1002/cssc.202401968

**Published:** 2024-11-07

**Authors:** Que Thi Nguyen, Jun Young Lee, Yejin Bae, Yu‐Ri Lee, Younghan Song, Sang Hoon Kim, Kyung‐Youl Baek, Jongbeom Na

**Affiliations:** ^1^ Materials Architecturing Research Center Korea Institute of Science and Technology Seoul 02792 Republic of Korea; ^2^ Extreme Materials Research Center Korea Institute of Science and Technology Seoul 02792 Republic of Korea; ^3^ Division of Nanoscience and Technology KIST School University of Science and Technology Daejeon 34113 Republic of Korea; ^4^ Department of Energy Engineering Hanyang University Seoul 04763 Republic of Korea; ^5^ Greenhouse Gas Research Laboratory Korea Institute of Energy Research Daejeon 305–343 Republic of Korea; ^6^ KHU-KIST Department of Converging Science and Technology Kyung Hee University Seoul 02447 Republic of Korea; ^7^ Australian Institute for Bioengineering and Nanotechtnology (AIBN) The University of Queensland Brisbane, Queensland 4072 Australia

**Keywords:** Adsorption, Mesoporous materials, Metal-organic framework, Multifunctional groups, ZIF-8

## Abstract

This study demonstrated a simple and innovative way of using the direct *de novo* synthesis to fabricate the mesoporous structure and diverse functionality of ZIF‐8 for environmental cleanup and gas storage applications. By introducing different ligands, we have developed a version of ZIF‐8 that could better capture carbon dioxide (CO_2_) and iodine. The ZIF‐8 was successfully designed to have the hierarchical and mesoporous structure with the functional groups of amine and thiol groups by adjusting the pKa values (from 8 to 12) of ligand instead of the original ligand, 2‐methyl imidazole (Hmim, pK_a_~14.2). The modulation of ZIF‐8 particle size, porosity, and functional characteristics was achieved through varied ligands and their concentrations, streamlined into a single and room‐temperature synthesis condition. The resulting ZIF‐8 materials exhibit intricate hierarchical architectures and a high density of functional groups, significantly enhancing molecular diffusion and accessibility. Among the developed materials, ZIF‐8‐AS, featuring both amine and thiol groups, demonstrates the fastest adsorption kinetics and a twofold increase in iodine adsorption capacity (q_m_=1101.5 mg g^−1^) compared to ZIF‐8 (q_m_=514.3 mg g^−1^). Furthermore, the hierarchical mesoporosity of ZIF‐8‐A‐10.1 improves CO_2_ adsorption to 1.0 mmol g^−1^ at 298 K, which is 1.3 times higher than that of the microporous ZIF‐8.

## Graphical abstract

This study reports the synthesis of hierarchical mesoporous ZIF‐8 with diverse functionalities using a *de novo* mixed ligand synthesis strategy. By adjusting the ligand properties, hierarchical structures containing with amine and thiol groups were achieved, enhancing CO_2_ capture and iodine adsorption. The straightforward method manipulated particle size and structure, offering a scalable, room‐temperature synthesis process with significant industrial potential.

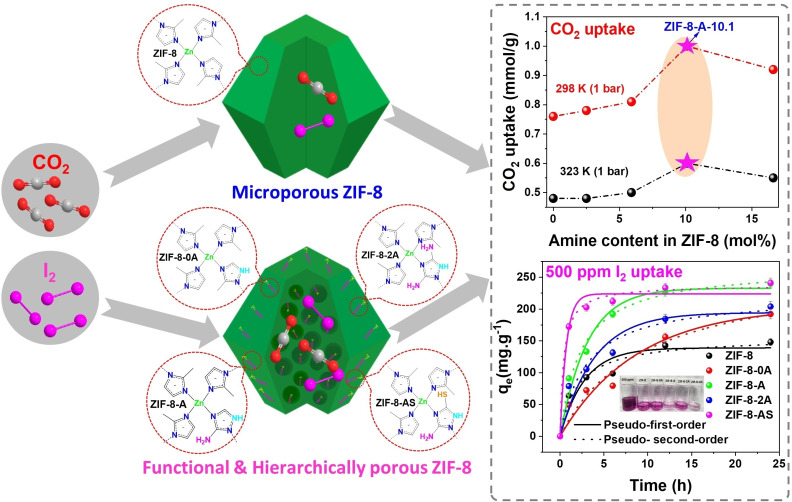

## Introduction

The increasing levels of carbon dioxide in the atmosphere represent a significant environmental concern, as CO_2_ is the primary greenhouse gas driving global warming.[^1^, ^2^] Consequently, global warming has become a critical ecological issue that poses a threat to human survival and development. Additionally, radiological iodine isotopes like ^129^I and ^131^I, found in nuclear waste, pose serious risks to both health and the environment due to their long half‐lives, high volatility, and strong potential for spreading in water.[^3^, ^4^, ^5^, ^6^] Therefore, developing effective adsorbents with high adsorption capacity, low cost, and robust stability for capturing radioactive iodine and CO2 is crucial to address these global challenges.

Zeolitic imidazolate frameworks‐8 (ZIF‐8) consist of tetrahedrally coordinated Zn metal ions and imidazole linkers, a subclass of microporous metal‐organic frameworks (MOFs),[^7^, ^8^] has gained considerable attention due to its large surface area, excellent physical stability, chemical reactivity, tunable structure and porosity for potential applications in gas adsorption, separation, catalysis, etc.^[9]^ ZIF‐8 has demonstrated a good candidate to capture radioactive iodine and CO_2_ due to the large cavities (11.6 Å) and small pore windows (3.4 Å), which closely match the kinetic diameter of iodine molecules (3.35 Å) and CO_2_ (3.3 Å) and the excellent thermal stability.[^3^, ^10^, ^11^, ^12^, ^13^] However, the inherently weak adsorption sites on the pore surfaces of ZIF‐8 limit its sorption capacity for CO_2_ and I_2_.[^3^, ^13^] To improve its adsorption performance, the functionalization of ZIF‐8 is considered essential. The combination of metal ions and ligands in ZIF‐8 enhances its versatility, making it a suitable platform for tailoring specific functionalities. Therefore, adjusting the functional groups and pore structure of microporous ZIF‐8 has significant potential for enhancing performance. This approach can overcome limitations related to low diffusion effects and fewer active sites of ZIF‐8, thereby expanding their applicability and effectiveness in various applications. This quality renders it an attractive candidate for CO_2_ and iodine adsorption applications. The synthesis of functional and hierarchical porous ZIF‐8 with micro‐mesoporous features greatly enhances its applicability. Incorporating specific functional groups into ZIF‐8 presents a promising approach for improving its performance. Researchers have explored reinforcing ZIF stability through post‐synthetic linker‐exchange (PSLE) using multiple ligands.[^14^, ^15^, ^16^, ^17^, ^18^, ^19^, ^20^, ^21^, ^22^] This thermodynamic approach allows for synthesizing ZIFs with desired properties and functional groups. In previous studies,[^3^, ^13^] the synthesis of amine‐functionalized ZIF‐8 (ZIF‐8‐A) with 3‐amino‐1,2,4‐triazole (A) via post‐synthetic ligand exchange demonstrated significantly higher CO_2_ and iodine adsorption compared to the original ZIF‐8. This enhancement can be attributed to increased chemical interactions with CO_2_ and iodine, facilitated by free amine groups acting as Lewis bases and the polar nature of the 1,2,4‐triazole ring, along with abundant N‐groups. However, post‐synthetic ligand exchange methods involve multiple steps and specific temperatures, gradually reducing surface area and pore volume in modified ZIF‐8. It may restrict their scalability for large‐scale synthesis, making them less suitable for industrial applications. In another approach, the vapor‐phase ligand exchange (VPLE)^[23]^ can introduce different imidazole derivatives but is limited to specific linkers and may cause phase transformation. To address this limitation, a straightforward and more scalable method is required to directly incorporate ligands that introduce functional groups into the ZIF‐8 framework. This strategy ensures the preservation of its high surface area and pore structure to enhance molecule accessibility. For such kind of synthesis, it is crucial to select the linker to retain the topology and structure of ZIF‐8. Some reports also highlighted the benefits of functionalized ZIF‐8 as active sites for CO_2_ adsorption through the synthesis of direct mixed dual‐ligands. For example, Zhang et al.^[24]^ synthesized MAF‐7 using mixed ligands of 3‐methyl‐1,2,4‐trizole and 2‐methyl imidazole (Hmim), exhibiting tunable ZIF‐8 properties. MAF‐7 exhibited significantly enhanced CO_2_ adsorption performance of 62.5 m^3^/g compared to ZIF‐8 (also named MAF‐4) of 29.3 cm^3^ g^−1^ at 273 K. The uncoordinated N donor in the triazole linker in MAF‐7 plays a role of Lewis base active sites, promoting dipole‐quadrupole interactions with the carbon atom of CO_2_. Eum et al.^[25]^ reported mixed‐linkers to synthesize ZIF‐8–90 by *in situ* incorporation of carboxaldehyde‐2‐imidazole (ZIF‐90 linker) and Hmim linker for adjustable framework functionality, allowing for the tuning of molecular sieving properties such as pore size, hydrophilicity, and organophilicity, as well as tuning the adsorption behavior. Thompson et al.^[26]^ modified the surface of ZIF‐8 with tailored amino groups using a mixed‐linker synthesis approach, incorporating 2‐aminobenzimidazole as a substitution linker to enhance adsorption selectivity for CO_2_/CH_4_ and heat adsorption for CO_2_.

In addition, the hierarchically micro‐ and mesoporous structure of MOFs offer distinct advantages. Microporosity provides a high surface area and great pore volumes, while mesoporosity could enhance diffusion by reducing the transport resistance of molecular diffusion and providing better accessibility of guest molecules to the framework.[^27^, ^28^, ^29^] Therefore, to address this diffusion limitation of microporous ZIF‐8, the hierarchical mesoporosity into microporous ZIF‐8 crystals becomes an intriguing strategy to enhance mass transfer kinetics.^[30]^ So far, various approaches, including templating, etching, and thermal treatment, have been proposed to enlarge the pore size of ZIF‐8.[^31^, ^32^, ^33^] However, these methods have some drawbacks, such as unfavorable conditions such as elevated temperatures and prolonged reaction durations. In addition, the selection of surfactant templates should be tailored specifically to the types of MOFs.^[34]^ The use of sacrificial templates and the growth of MOFs on those templates require removal conditions to avoid defects or polycrystalline structures.[^35^, ^36^] This process has limitations, such as the inability to create single crystals and complications in removing the template, structural collapse, and residue of the template after removal. Recently, Huang et al.^[37]^ reported the creation of hierarchically porous ZIF‐8 through selective cleavage of thermolabile ligands, specifically 2‐aminobenzimidazole (NH_2_‐bIm), by controlling thermal conditions in the mixed ligands for ZIF‐8. This mixed‐ligand ZIF‐8 was synthesized via a solvent‐assisted ligand exchange method between ZIF‐8 and NH_2_‐bIm ligand. The development of hierarchically micro‐ and mesoporous ZIF‐8 led to a 40‐fold increase in methylene blue dye adsorption compared to pristine ZIF‐8. This enhancement can be attributed to the hierarchically mesopore architecture, facilitating the accelerated diffusion of dye molecules to active sites. However, it is important to note that this method is a complicated process that consumes energy and requires carefully controlled thermal temperatures to remove the thermolabile ligand. This could potentially lead to the collapse of the ZIF‐8 structure, damage to hierarchical pores, and oxidation of amine groups during heat treatment. Additionally, it might result in a reduction in the surface area of ZIF‐8 and low quality of hierarchical mesoporous formation in ZIF‐8. These alternative methods typically necessitate templates or complicated procedures, which challenges their application in different systems and complicates scaling up the process. Thus, it is desirable to develop a breakthrough method for synthesizing hierarchically mesoporous ZIF‐8 materials in a simple and low‐cost manner for large‐scale applications.

Considering the aforementioned factors, we present a novel approach for synthesizing hierarchical mesoporous ZIF‐8 with functional amine and thiol groups. This method employs a mixed‐ligand approach, offering a direct and straightforward synthesis route that is typically difficult to achieve using conventional methods. By introducing four different ligands, specifically 1,2,4‐triazole (0 A), 3‐amino‐1,2,4‐triazole (A), 3,5‐diamino‐1,2,4‐triazole (2 A), and 3‐amino‐1,2,4‐triazole‐5‐thiol (AS), which feature bulkier substituents at specific positions, along with 2‐methylimidazolate coordinated with Zn(II) metal clusters, we successfully synthesized four distinct crystalline compounds: ZIF‐8–0 A, ZIF‐8‐A, ZIF‐8–2 A, and ZIF‐8‐AS, respectively.

A precisely controlled hierarchy of mesoporous ZIF‐8 with varying mesopore sizes, particle sizes, and multiple functional groups (amine and thiol) was achieved through the use of mixed multivariate linkers containing heterocyclic nitrogen‐containing five‐membered rings with different pKa values. The particle size and mesopore size were regulated by adjusting the concentration and type of mixed linkers. Additionally, the mechanism behind the formation of mesoporosity in ZIF‐8 was investigated. These compounds exhibit phase‐pure and well‐defined crystalline properties, overcoming the challenges associated with synthesizing ZIF‐8 using multiple ligands. The hierarchical porous (mesoporous) ZIF‐8‐AS, enriched with abundant amine and thiol groups, offers numerous active sites for iodine adsorption, resulting in a twofold increase in iodine adsorption capacity (q_m_ = 1101.5 mg g^−1^) compared to pristine microporous ZIF‐8. This enhancement is crucial for various industrial applications, environmental remediation, and nuclear waste management. A hierarchically mesoporous ZIF‐8‐A, featuring multiple amine groups as chemisorption active sites and an abundant heterocyclic nitrogen‐containing five‐membered ring, was employed for CO_2_ adsorption. It exhibited higher CO_2_ uptake compared to pristine ZIF‐8, due to the synergistic effect of the hierarchical mesoporous structure and multiple chemically active sites. Specifically, ZIF‐8‐A‐10.1, which has the highest hierarchical mesoporosity, increased CO_2_ adsorption capacity to 1.0 mmol g^−1^ at 298 K, which is 1.3 times higher than that of microporous ZIF‐8. These synthesized ZIFs show promise as materials for iodine and CO_2_ uptake applications, addressing the pressing challenges associated with greenhouse gas emissions and nuclear waste management.

## Experimental Section

### Synthesis of Microporous ZIF‐8

ZIF‐8 was synthesized following the basis of the literatures[^38^, ^39^] with a little modification. The amount of chemical reagents used is listed in Table S1. Firstly, 4.76 g of Zn(NO_3_)_2⋅_6H_2_O and 10.51 g of linker Hmim were dissolved separately in 320 mL and 160 mL methanol (MeOH), respectively, under stirring for 30 minutes at room temperature. The metal salt mixture was added to the ligand mixture under stirring for 24 h. The white precipitate product was collected by centrifugation at 10000 rpm and then washed with methanol several times. Finally, ZIF‐8 was dried in a vacuum at 100 °C for 24 h.

### Synthesis of a Functional & Hierarchical Porous ZIF‐8–0 A, ZIF‐8‐A, ZIF‐8–2 A, ZIF‐8‐AS

The amount of chemical reagent used to synthesize functional & hierarchical porous ZIF‐8–0 A, ZIF‐8‐A, ZIF‐8–2 A, and ZIF‐8‐AS materials are summarized in Table S1. The ligand mixture, containing a total of 128 mmol of Hmim and linker II (1,2,4‐triazole (0 A), 3‐ amino 1,2,4‐triazole (A), 3,5 diamino‐1,2,4‐triazole (2 A), 3‐amino‐1,2,4‐triazole‐5‐thiol (AS), with a molar ratio of linker Hmim:linker II (98 : 2) in MeOH, was added to a metal solution containing 16 mmol of Zn(NO_3_)_2⋅_6H_2_O in MeOH. This mixture solution was kept under stirring at room temperature for 24 h. The resulting products were isolated through centrifugation and multiple washes with MeOH and then dried at 100 °C under vacuum conditions overnight. Based on the second linker II (0 A, A, 2 A, AS) used in synthesis, the obtained products were denoted as ZIF‐8–0 A, ZIF‐8‐A, ZIF‐8–2 A, and ZIF‐8‐AS, respectively.

### Synthesis of Amine Functional and Hierarchical Porous ZIF‐8‐A with Different Concentration of A Linker

The amounts of chemical reagents used to synthesize functional and hierarchical ZIF‐8‐A materials were summarized in Table S2. The ligand mixture, containing a total of 128 mmol of Hmim and 3‐ amino 1,2,4‐triazole (A) at various molar ratios of Hmim:A (99.57:0.43, 99 : 1, 98 : 2, 96.8 : 3.2) in MeOH, was added to a metal solution containing 16 mmol of Zn(NO_3_)_2⋅_6H_2_O in MeOH. Following a procedure similar to that used for synthesizing ZIF‐8‐A but varying the content of the A ligand, the obtained products were denoted as ZIF‐8‐A‐n (ZIF‐8‐A‐2.5, ZIF‐8‐A‐5.9, ZIF‐8‐A‐10.1, ZIF‐8‐A‐16.6), where n=(2.5, 5.9, 10.1, 16.6) represents the mole fraction of linker A in the final material, respectively.

### Iodine Adsorption and CO_2_ Adsorption

The experimental details for iodine and CO_2_ adsorption are provided in the experimental section in SI.

### Characterization

The details of the characterization for the resulting ZIF‐8 are provided in SI.

## Result and Discussion

### Effect of Various Linkers on Functional & Hierarchical Porous ZIF‐8

**Figure 1:**
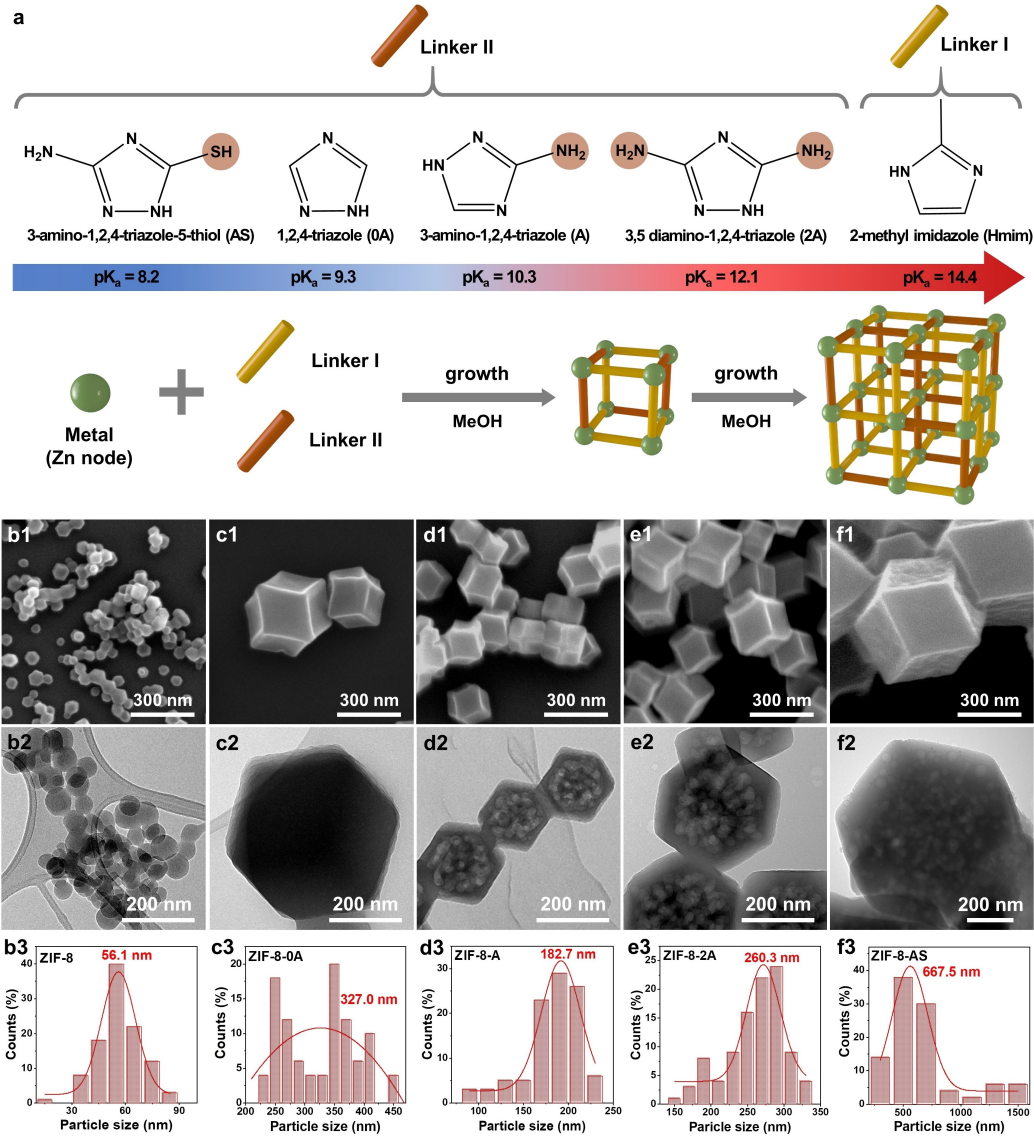
(a) Scheme of synthesis of microporous ZIF‐8 and functional & hierarchical porous ZIF‐8‐II with mixed multivariate ligands. SEM images of (b1) ZIF‐8, (c1) ZIF‐8–0 A, (d1) ZIF‐8‐A, (e1) ZIF‐8–2 A, and (f1) ZIF‐8‐AS materials. TEM images of (b2) ZIF‐8, (c2) ZIF‐8–0 A, (d2) ZIF‐8‐A, (e2) ZIF‐8–2 A, and (f2) ZIF‐8‐AS materials. Particle size distribution calculated from SEM images of (b3) ZIF‐8, (c3) ZIF‐8–0 A, (d3) ZIF‐8‐A, (e3) ZIF‐8–2 A, and (f3) ZIF‐8‐AS materials.

The dual‐functional and hierarchical porous (mesoporous) ZIF‐8 was synthesized by mixing a combination of dual ligands (Hmim and Linker II (0 A, A, 2 A, AS)) with Zn (NO_3_)_2_.6H_2_O salt in methanol at room temperature for 24 hours as illustrated in Figure 1a. This process involved incorporating various functional groups into the ZIF‐8 framework by using distinct linkers with 2‐methylimidazole. These groups include one or two free amine (−NH_2_) and thiol (−SH) groups alongside N‐rich heterocyclic rings in the triazole structure. This strategy enabled the adjustment of the physical and chemical attributes of the ZIF‐8 framework, encompassing its structural morphology, particle dimensions, and stratified mesoporosity. Consequently, this enhancement optimized the molecular interactions attributed to the functional groups and the nitrogen‐enriched heterocyclic triazole ring.

Table 1 summarizes the amounts of linker II incorporated into ZIF‐8‐II frameworks, which were characterized using ^1^H‐NMR and ^13^C‐NMR analyses (Figure S1–S3). The correlation between the initial amount of linker II in the synthesis solution and the final amount of linker II in the resulting products of ZIF‐8‐II frameworks is depicted in Figure S4–S7. In the synthesis of ZIF‐8–0 A, an initial solution containing a mix of dual linkers 2 mol % of the 0 A linker and 98 mol % of the Hmim linker yielded a final product with a composition of 10.7 mol % 0 A linker and 89.3 mol % Hmim linker within the framework. Similarly, the synthesis of ZIF‐8‐II (ZIF‐8‐A, ZIF‐8–2 A, and ZIF‐8‐AS) starting from a solution with only 2 mol % of linker II (comprising A, 2 A, AS) and 98 mol % Hmim, resulted in final products incorporating 10.1 mol % A, 9.2 mol % 2 A, and 11.5 mol % AS, respectively. These results indicate a pronounced preference for linker II incorporation into the ZIF‐8‐II framework over the Hmim linker. This preference is attributed to the greater number of nitrogen (N) vacancies in linker II, which range from 3 to 5 N atoms, compared to the Hmim linker, which contains only 2 N atoms. The increased number of N atoms in linker II likely facilitates enhanced coordination with the Zn metal nodes within the framework. The concentration in those synthetic conditions did not change the original ZIF‐8 crystalline structure, as confirmed by X‐ray diffraction (XRD) patterns. The maximum amounts of the liker 0 A, A, 2 A, and AS in the ZIF‐8 framework to preserve the crystalline structure of ZIF‐8 were 67.0 mol %, 46.8 mol %, 9.2 mol %, and 11.5 mol %, respectively. The phase transformation and amorphous structures of ZIF‐8‐II were observed above these concentrations.

**Table 1:** Physical properties of ZIF‐8 and ZIF‐8‐II.

Samples	S_BET_^[a]^ (m^2^ g^−1^)	V_pore_^[b]^ (cm^3^ g^−1^)	Particle size^[c]^ (nm)	Amount of II^[d]^ (mol %)	Average mesoporous^[e]^ (nm)	Crystallite size^[f]^ (nm)	Crystallinity^[g]^ (%)
ZIF‐8	1758	0.771	56.9	0	0	48.60	87.8
ZIF‐8–0 A	1738	0.666	327.0	10.7	0	77.21	85.4
ZIF‐8‐A	1583	0.712	182.7	10.1	75.1	68.97	84.8
ZIF‐8–2 A	1614	0.634	260.3	9.2	51.9	70.62	85.3
ZIF‐8‐AS	961	0.476	667.5	11.5	39.8	81.64	82.6

[a] BET surface area determined by N_2_ adsorption‐desorption isotherm at 77 K; [b] Total pore volume; [c] Determined from SEM; [d] Calculated from ^1^H‐NMR and ^13^C‐NMR analysis (DMSO/H_2_SO_4_=9/1, v/v); [e] Average size of mesopores calculated from TEM imagine; [f] Determined from Scherrer equation (XRD); [g] Calculated from XRD.

The morphologies of the prepared samples were displayed in Figure 1, and the mean size of ZIF‐8 particles were summarized in Table 1. The morphology of the pristine ZIF‐8 exhibited a rhombic dodecahedron with a particle size of about 56.9 nm. The morphological characteristics of functional and hierarchical porous ZIF‐8‐II were influenced by the second linker (linker II). ZIF‐8–0 A, ZIF‐8‐A, ZIF‐8–2 A, and ZIF‐8‐AS exhibited a rhombic dodecahedra morphology shape. The particle sizes increased in the following order: ZIF‐8 (56.9 nm) < ZIF‐8‐A (182.7 nm) < ZIF‐8–2 A (260.3 nm) < ZIF‐8–0 A (327.0 nm) < ZIF‐8‐AS (626.6 nm). Interestingly, a discernible trend was identified in which ligands with higher pKa values were associated with forming smaller particle sizes, as demonstrated in Figure S8a. This observation may be attributed to the effect of the protonation state of the second ligand on both the nucleation and growth rates during the ZIF‐8 formation process. An exception to this trend was noted in the comparison between ZIF‐8–2 A and ZIF‐8‐A, where ZIF‐8–2 A exhibited larger particle sizes than ZIF‐8‐A despite the higher pKa value of linker 2 A (pKa ~12.1) relative to linker A (pKa ~10.3). This phenomenon could be explained by the increased steric hindrance and enhanced coordination capability of the 2 A linker, attributable to its two free amine groups. Such characteristics likely promote higher growth rates and aggregation, leading to the observed larger particle size in ZIF‐8–2 A. In the case of ZIF‐8‐AS, the larger particle size can be reasoned with the more pronounced steric hindrance and the more acidic nature (evidenced by the lowest pKa) of the AS ligand, which may inhibit nucleation in ZIF‐8‐AS.

**Figure 2:**
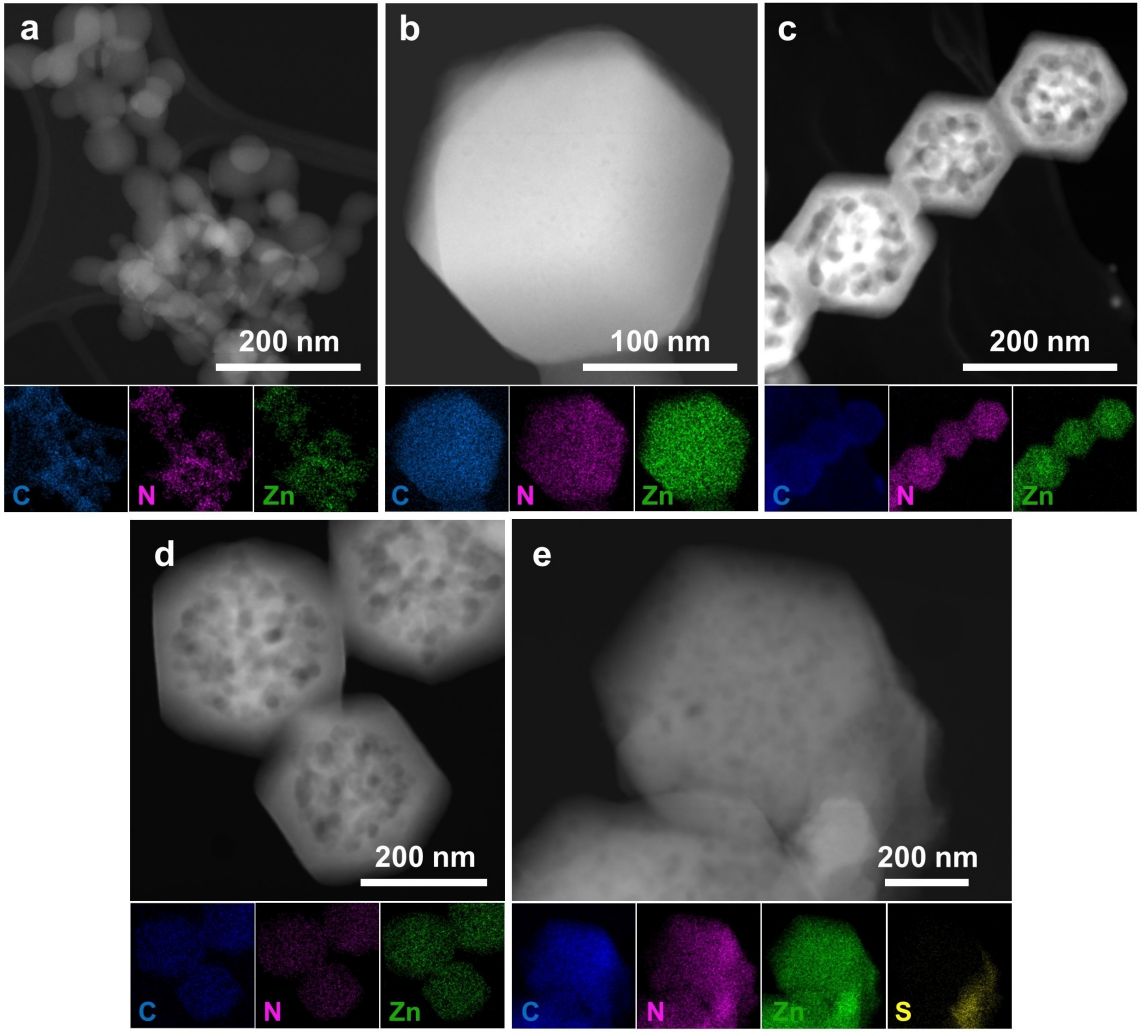
HAADF‐STEM imagine corresponding with elemental (C, N, Zn, S) EDX mapping of (a) ZIF‐8, (b) ZIF‐8–0 A, (c) ZIF‐8‐A, (d) ZIF‐8–2 A, (e) ZIF‐8‐AS materials.

Transmission electron microscopy (TEM) images, high‐angle annular dark‐field scanning transmission electron microscopy (HAADF‐STEM) images, and energy‐dispersive X‐ray spectroscopy (EDX) elemental mapping are presented in Figures 1 and 2. ZIF‐8 was observed to have a smooth surface devoid of mesopores alongside a homogeneous distribution of Zn, C, and N elements. EDX elemental mapping revealed the uniform presence of C, N, and Zn elements in ZIF‐8–0 A, ZIF‐8‐A, ZIF‐8–2 A, and ZIF‐8‐AS. Notably, ZIF‐8‐AS also displayed the additional presence of the S element, suggesting the successful incorporation of the second ligand (linker II) into the ZIF‐8‐II framework, as shown in Figure 2. This observation is consistent with the XPS data (Figure S9, Table S3), which reveals a new peak for S that appeared compared to ZIF‐8, which lacks S, with 2.22 % atomic S in ZIF‐8‐AS. TEM images indicated mesoporosity within the ZIF‐8‐II materials, with pore sizes ranging from 10 nm to 80 nm (referenced in Figure 1, S8b, and Table 1). Specifically, the ZIF‐8–0 A material exhibited exclusively microporosity, devoid of mesopores, while ZIF‐8‐A displayed a hierarchical mesoporous structure with an average mesopore size of 75.1 nm. A hierarchical mesoporous structure was also observed in ZIF‐8–2 A, with an average pore size of 51.9 nm. In contrast, ZIF‐8‐AS featured a mesoporous structure with an average pore size of 39.8 nm, albeit with a lower population of pores.

Among the prepared samples, ZIF‐8‐A was observed to exhibit the highest level of mesoporous structure and the largest average pore size. The formation of mesoporosity in the ZIF‐8‐II series is likely to occur through the cleavage and migration of linker II during the coordination process of the competitive ligand. This involves a simultaneous ligand exchange and reassembly of the two ligands, contributing to the development of mesoporosity. Consequently, ZIF‐8‐A, which displayed the most pronounced hierarchical mesoporous structure among the synthesized materials, was selected for further investigation into the effect of the concentration of linker A on the hierarchical mesoporous structure of ZIF‐8.

XRD patterns, along with corresponding crystallinity and crystallite sizes of these materials, are depicted in Figures S10, S11, and Table 1. The prepared ZIF‐8 displayed characteristic diffraction peaks identical to those reported previously, confirming the successful synthesis of ZIF‐8.^[40]^ The XRD patterns of ZIF‐8‐II showed peaks similar to pristine ZIF‐8, indicating that the incorporation of the second ligand (linker II) preserved the crystal structure of ZIF‐8. However, these peaks shifted towards higher angles, indicating a decrease in d‐spacing, suggesting a more compact structure in ZIF‐8‐II than pristine ZIF‐8. This observation is in line with previous reports.[^41^, ^42^] Moreover, the introduction of second ligands into ZIF‐8 maintained high crystallinity, with ZIF‐8‐II exhibiting over 80 % crystallinity, slightly lower than that of pristine ZIF‐8 (87.8 %). The relationship between crystallite size (as determined from XRD patterns) and the pKa value of linker II in ZIF‐8‐II is detailed in Figure S11. The sequence of crystallite sizes for different linkers in ZIF‐8‐II follows the order: ZIF‐8 (48.6 nm) < ZIF‐8‐A (68.97 nm) < ZIF‐8–2 A (70.62 nm) < ZIF‐8–0 A (77.21 nm) < ZIF‐8‐AS (81.64 nm), a trend that is consistent with particle size observations from SEM images.

The Fourier transform infrared (FT‐IR) spectra (Figure S10b) of all samples showed a consistent peak around 421 cm^−1^, attributed to the coordinated Zn−N (Zn from metal node and N from Hmim ligand) in pristine ZIF‐8.^[43]^ The relatively low content of ligand II in the ZIF‐8‐II frameworks, at 10 mol % compared to the Hmim ligand, likely contributed to the negligible alteration in the Zn−N stretching vibration in ZIF‐8‐II when compared to pristine ZIF‐8. Furthermore, the peaks identified within the spectral range of 3200–3600 cm^−1^ were indicative of functional groups, including ‐OH, ‐NH, and ‐NH_2_ groups.

The BET surface area of ZIF‐8‐II was illustrated in Figure S10c and summarized in Table 1. ZIF‐8 exhibited a high surface area of 1758 m^2^ g^−1^ and a pore volume of 0.771 cm^3^ g^−1^. In comparison, other samples showed a surface area in the range of 900–1800 m^2^ g^−1^ and a high pore volume of approximately 0.5–0.7 cm^3^ g^−1^. These results suggested that introducing a second ligand can maintain the high surface area and pore volume characteristics of ZIF‐8.

Figure S10d exhibited the weight loss versus temperature curves of the samples under air atmosphere to demonstrate the impact of functional groups on the thermal stability behavior of ZIF‐8‐II materials. The thermal stability of the ZIF‐8‐II was greatly dependent on the second organic linkers. The ZIF‐8, ZIF‐8–0 A, and ZIF‐8‐A followed similar weight loss curves, but the ZIF‐8–2 A and ZIF‐8‐AS showed no similarity. It may be attributed to the accelerated thermal decomposition and oxidation by the functional groups of organic linkers.[^44^, ^45^, ^46^, ^47^] The functional group with sulfur and nitrogen‐containing elements with bulkier structure may accelerate decomposition and oxidation. Therefore, the thermal stability was dependent on order at 350 °C: ZIF‐8 > ZIF‐8–0 A > ZIF‐8‐A > ZIF‐8–2 A > ZIF‐8‐AS.

### Effect of Concentration of Linker A on Functional & Hierarchical Porous of ZIF‐8

**Figure 3:**
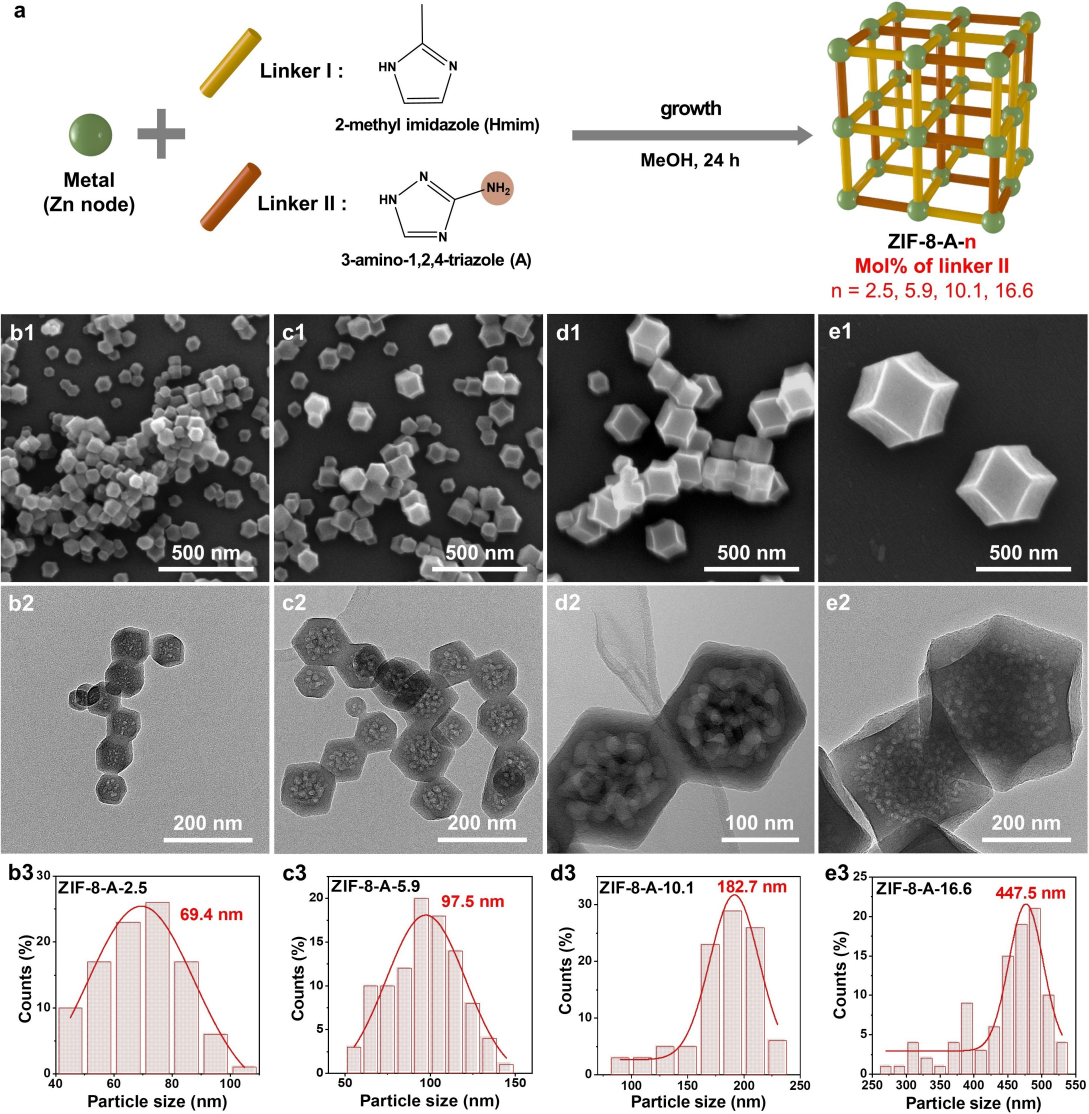
(a) Scheme of synthesis of amine functional & hierarchical porous ZIF‐8‐A materials with different concentrations of linker A. SEM images of (b1) ZIF‐8‐A‐2.5, (c1) ZIF‐8‐A‐5.9, (d1) ZIF‐8‐A‐10.1, (e1) ZIF‐8‐A‐16.6 materials. TEM images of (b2) ZIF‐8‐A‐2.5, (c2) ZIF‐8‐A‐5.9, (d2) ZIF‐8‐A‐10.1, (e2) ZIF‐8‐A‐16.6 materials. Particle size distributions (calculated from SEM images) of (b3) ZIF‐8‐A‐2.5, (c3) ZIF‐8‐A‐5.9, (d3) ZIF‐8‐A‐10.1, (e3) ZIF‐8‐A‐16.6 materials.

**Figure 4:**
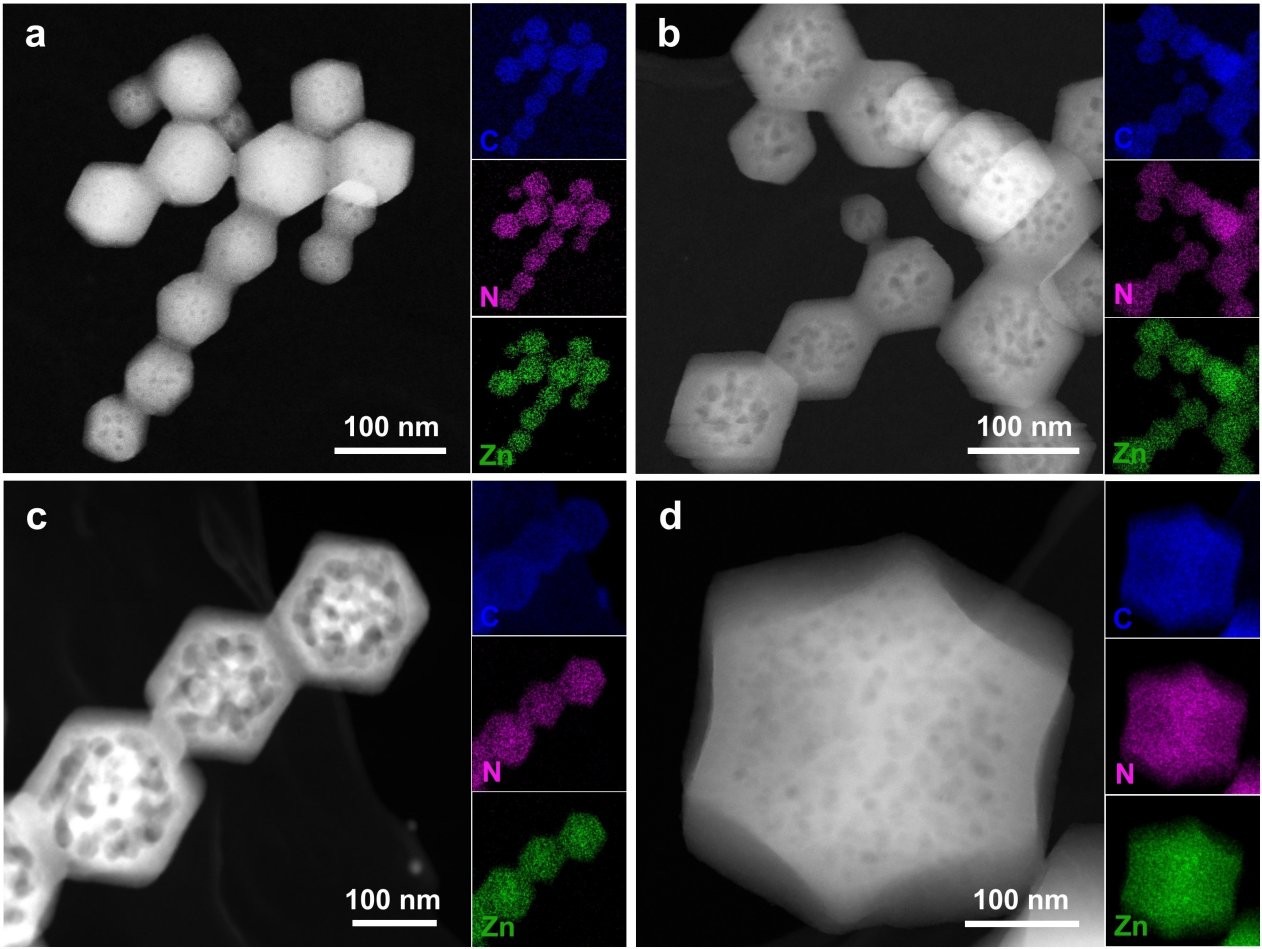
HAADF‐STEM imagine corresponding with elemental (C, N, Zn) EDX mapping of (a) ZIF‐8‐A‐2.5, (b) ZIF‐8‐A‐5.9, (c) ZIF‐8‐A‐10.1, (d) ZIF‐8‐A‐16.6 materials.

Figure 3a illustrates the synthesis process of the amine‐functionalized and hierarchical mesoporous ZIF‐8‐A series, wherein varying amounts of linker A were incorporated into ZIF‐8‐A. To synthesize ZIF‐8‐A, a mixture of Hmim and linker A in MeOH was introduced to the Zn solution, resulting in ZIF‐8‐A formation. The impact of 3‐amino‐1,2,4‐triazole (A) ligand concentration on ZIF‐8 formation was studied. With an increase in the concentration of the second ligand (A), the particle size gradually increased. For example, the particle size ranged from 56.9 nm for ZIF‐8 with 0 mol % of linker A to 447.5 nm for ZIF‐8‐A‐16.6 with 16.6 mol % of linker A, as observed in SEM images and particle size distribution analysis (Figures 3, S12, and Table 2). ZIF‐8‐A maintains a rhombic dodecahedra morphology akin to ZIF‐8, with the augmented particle size potentially stemming from the elevated N vacancies in the A ligand relative to the Hmim ligand. As suggested in the previous study,^[48]^ these four N atoms in second ligand A may preferentially coordinate more with Zn metal nodes, and the potentially aggregation‐prone nature, due to the relatively unstable surface of ZIF‐8‐A, may lead to an increased particle size ZIF‐8‐A compared to pristine ZIF‐8. HAADF‐STEM images and the corresponding EDX elemental mapping showed the uniform distribution of C, N, and Zn elements in a series of ZIF‐8‐A materials, as shown in Figure 4. Additionally, TEM and HAADF‐STEM images have exhibited the microporous and hierarchical mesoporous structure of ZIF‐8‐A (Figures 3 and 4). TEM images revealed mesoporous structures ranging from 8 nm to 80 nm in ZIF‐8‐A (Figures 3, S13, and Table 2). Even with a small amount of linker A (2.5 mol %) in ZIF‐8‐A, a hierarchical mesoporous structure emerged with an average size of 8.7 nm. With increasing amounts of linker A in ZIF‐8‐A up to 10.1 mol %, the population of hierarchical mesopores notably increased, reaching an average size of approximately 75.1 nm. However, further increasing the amount of linker A to 16.6 mol % resulted in a reduction in the mesoporous structure and average size. The highest population and size of mesoporous structures among these samples were observed for ZIF‐8‐A‐10.1.

**Table 2:** Physical properties of ZIF‐8‐A series.

Samples	S_BET_^[a]^ (m^2^ g^−1^)	V_pore_^[b]^ (cm^3^ g^−1^)	Particle size^[c]^ (nm)	Amount of A^[d]^ (mol %)	Average size of porous ^[e]^ (nm)	Crystallite size^[f]^ (nm)	Crystallinity^[g]^ (%)
ZIF‐8‐A‐2.5	1673	0.769	69.4	2.5	8.7	55.28	88.3
ZIF‐8‐A‐5.9	1583	0.713	96.4	5.9	17.6	58.01	87.8
ZIF‐8‐A‐10.1	1711	0.712	182.7	10.1	75.1	68.97	84.8
ZIF‐8‐A‐16.6	1579	0.661	447.5	16.6	16.2	71.49	90.1

[a] BET surface area determined by N_2_ adsorption‐desorption isotherm at 77 K; [b] Total pore volume; [c] Determined from SEM; [d] Calculated from ^1^H‐NMR analysis (DMSO/H_2_SO_4_=9/1, v/v); [e] Average size of mesopores calculated from TEM imagine; [f] Determined from Scherrer equation (XRD); [g] Calculated from XRD.

In Figure S14a, the XRD patterns of ZIF‐8‐A displayed sharp and similar peaks to those observed in pristine ZIF‐8, with no additional peaks present, indicating the high crystallinity and purity of the ZIF‐8 crystalline structure. The hierarchical mesoporous ZIF‐8‐A exhibited high crystallinity, ranging from 84 % to 90 %, compared to pristine ZIF‐8 (Table 2). The crystallite size of ZIF‐8‐A was calculated from XRD using the Scherrer equation, summarized in Table 2 and illustrated in Figure S15. With increasing linker A contents in ZIF‐8‐A from 0 to 16.6 mol %, the crystallite size also increased from 48.6 nm to 71.49 nm, consistent with the observed increase in particle size of ZIF‐8‐A in SEM images.

The concentration of linker A in the ZIF‐8‐A frameworks was quantified using ^1^H‐NMR spectroscopy, as shown in Figure S14b. The outcomes of this analysis were compiled in Table 2, which also includes plots demonstrating the correlation between the initial concentration of linker A in the synthesis solution and its subsequent integration into the ZIF‐8‐A framework, as shown in Figure S12. Notably, the actual mole ratio of linker A to the Hmim linker (A:Hmim) in the ZIF‐8‐A end products exceeded the mole ratio (A:Hmim) present in the initial synthesis mixture. This observation suggests an enhanced affinity and incorporation rate of ligand A into the ZIF‐8‐A framework compared to the Hmim linker, implying a preferential interaction of ligand A with the Zn metal nodes within the framework. For example, while the initial synthesis solution featured mole ratios (A:Hmim) of 0.43 : 99.57, 1 : 99, 2 : 98, and 3.2 : 96.8, the resulting ZIF‐8‐A products exhibited mole ratios (A:Hmim) of 2.5 : 97.5, 5.9 : 94.1, 10.1 : 89.9, and 16.6 : 83.4, respectively. This phenomenon indicates preferential coordination and a faster nucleation rate of ligand A with the zinc metal nodes over Hmim, likely due to ligand A's abundant nitrogen sites available for coordination and its lower pKa compared to Hmim.

FT‐IR spectra analysis of ZIF‐8‐A, detailed in Figure S14c, identified a peak at approximately 421 cm^−1^, indicative of Zn−N coordination bonds. Additionally, peaks within the 3200–3600 cm^−1^ range were associated with ‐NH, ‐NH_2_, and ‐OH functional groups. A distinct peak at about 1585 cm^−1^ corresponded to −C=N bonds, and newly emerged peaks at 1620 cm^−1^ were attributed to ‐NH_2_ groups, confirming the successful integration of linker A into the ZIF‐8‐A framework.

The BET surface area and pore volume for ZIF‐8‐A were depicted in Figure S14d and documented in Table 2. ZIF‐8‐A demonstrated a high surface area, approximately ranging from 1600 to 1800 m^2^ g^−1^, and a pore volume around 0.7–0.8 cm^3^ g^−1^. These values are comparable to those of pristine ZIF‐8, even at the highest tested concentration of linker A (16.6 %) in the ZIF‐8‐A frameworks. These results highlight the advantages of this synthetic approach over post‐synthetic ligand exchange methods in preserving the porosity of material.

Thermogravimetric analysis (TGA), presented in Figure S16, highlighted the high thermal stability of ZIF‐8‐A, with a degradation temperature of around 350 °C in air, akin to that of pristine ZIF‐8. The residue, primarily consisting of ZnO, accounted for approximately 35.5–36.4 % of the total weight at 700 °C, further illustrating the robustness of the ZIF‐8‐A framework.

### A Proposed Mechanism for the Formation of Hierarchical Mesoporous ZIF‐8 Structure

**Scheme 1:**
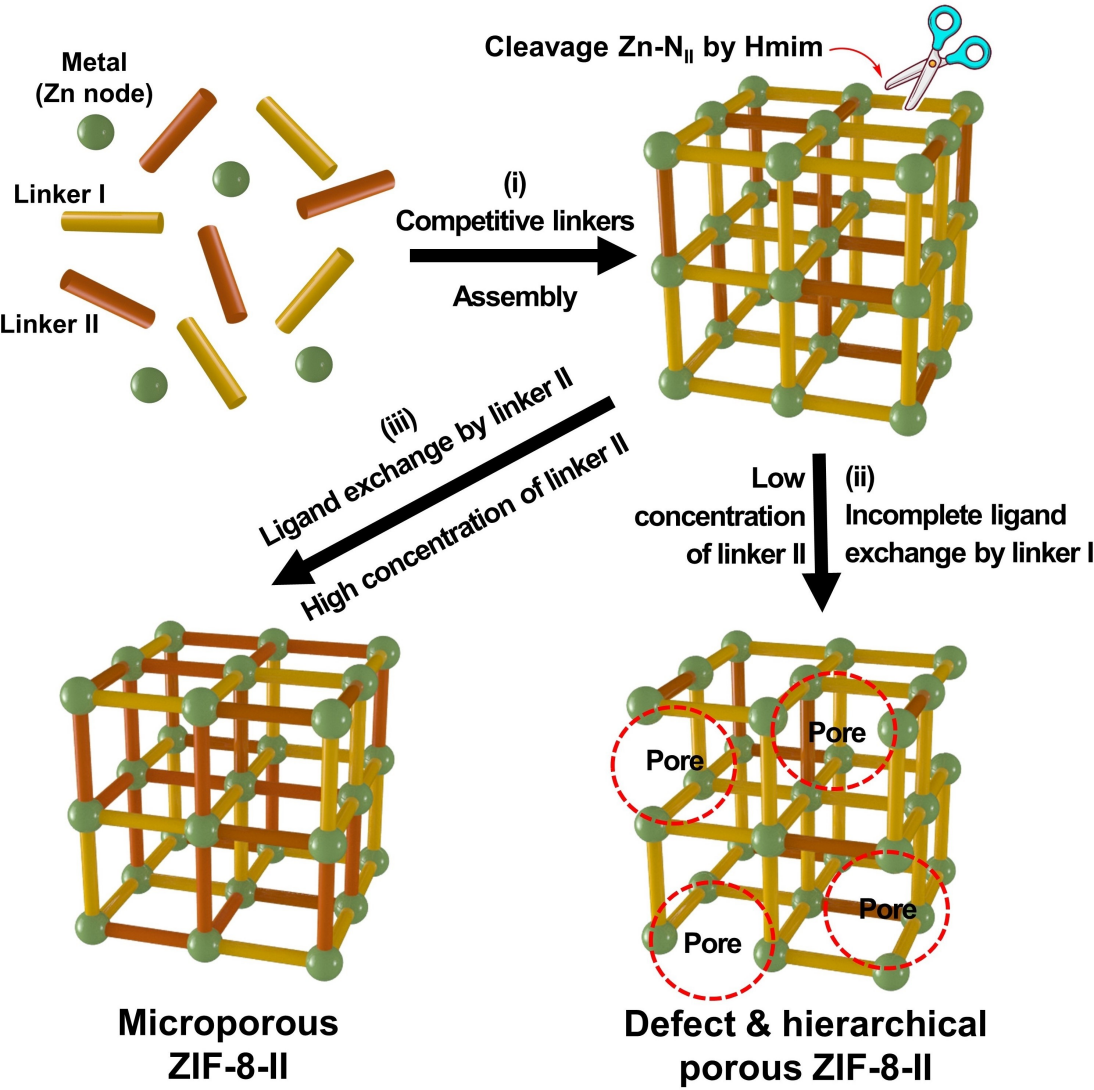
A Proposed mechanism for the hierarchical porous structure of ZIF‐8 through mixed ligand strategy.

In the synthesis process of ZIF‐8 with mixed linkers, several steps occur assembly, cleavage, exchange, migration, and reassembly. It can lead to the formation of microporous or hierarchical mesoporous ZIF‐8 structure, as shown in Scheme 1. In this process, linker II serves as a modulator and competitive ligand alongside Hmim, coordinating with the Zn metal cluster.^[49]^ The bulky groups in linker II could slow down ligand exchange process, resulting in incomplete exchange and creating structural defects and additional pore space. The higher pKa value of nitrogen‐based imidazole linkers leads to a more stable coordination between the ligand and metal. In contrast, linkers with lower basicity create weaker bonds, making them more susceptible to dissolution.[^50^, ^51^, ^52^] As a result, the lower basicity of ligand II results in the formation of less stable MOFs, whereas the higher basicity of Hmim contributes to the creation of more stable MOF networks. The presence of numerous nitrogen vacancies and the lower pKa value of linker II, which allows for easier deprotonation compared to Hmim, aids its coordination with the Zn node in forming ZIF‐8. This leads to a more rigid framework structure.[^53^, ^54^]

Post‐synthetic ligand exchange can be performed on ZIF‐8 using linker II (0 A, A, 2 A) and vice versa. This process is demonstrated to be reversible, as illustrated in Figures S17 and S18. The weaker coordination bonding between ligand II and Zn metal cluster compared to Hmim, allows for the cleavage of Zn‐N_II_ bonding (between Zn and Nitrogen from linker II) by Hmim and subsequent dissociation and release of linker II from the frameworks.^[55]^ This process leads to the reassembly of the framework structure with incomplete exchange ligand by Hmim linker, causing structural defects and a less rigid structure. The migration of ligand II could provide sites for further etching, enabling the creation of a mesoporous structure in ZIF‐8.[^56^, ^57^]

During the nucleation stage, the mixed ligands of Hmim and linker II competitively form ZIF‐8 seeds with zinc ions. At lower concentrations of linker II and higher concentrations of Hmim, there is a preferential cleavage of Zn‐N_II_ bonds by Hmim ligands. This mechanism facilitates the removal and replacement of linker II, leading to the formation of hierarchically mesoporous structures via a process characterized by incomplete ligand exchange and subsequent reassembly.

When there is a higher concentration of linker II, the equilibrium shifts towards exchanging Hmim ligands within the framework, promoting reassembly with linker II. This process culminates in the formation of microporous ZIF‐8 structures, distinguished by their more rigid architecture due to extensive coordination between nitrogen atoms in linker II and zinc clusters. Therefore, mesoporous structures are predominantly formed at lower concentrations of linker II, while higher concentrations lead to the emergence of microporous structures with larger particle sizes, attributed to their increased rigidity.

The critical role of linker II extends to etching and forming mesoporous structures; its absence in the synthesis process results in microporous ZIF‐8 structures due to the lack of a competitive ligand to facilitate coordination and etching processes. Several factors, including pKa values, the number of nitrogen atoms available for coordination, steric hindrance, and the concentration of linker II influence the hierarchical nature of the porous structure in ZIF‐8.

### Effect of Hierarchical Porous Structure of ZIF‐8‐A on CO_2_ Uptake

**Figure 5:**
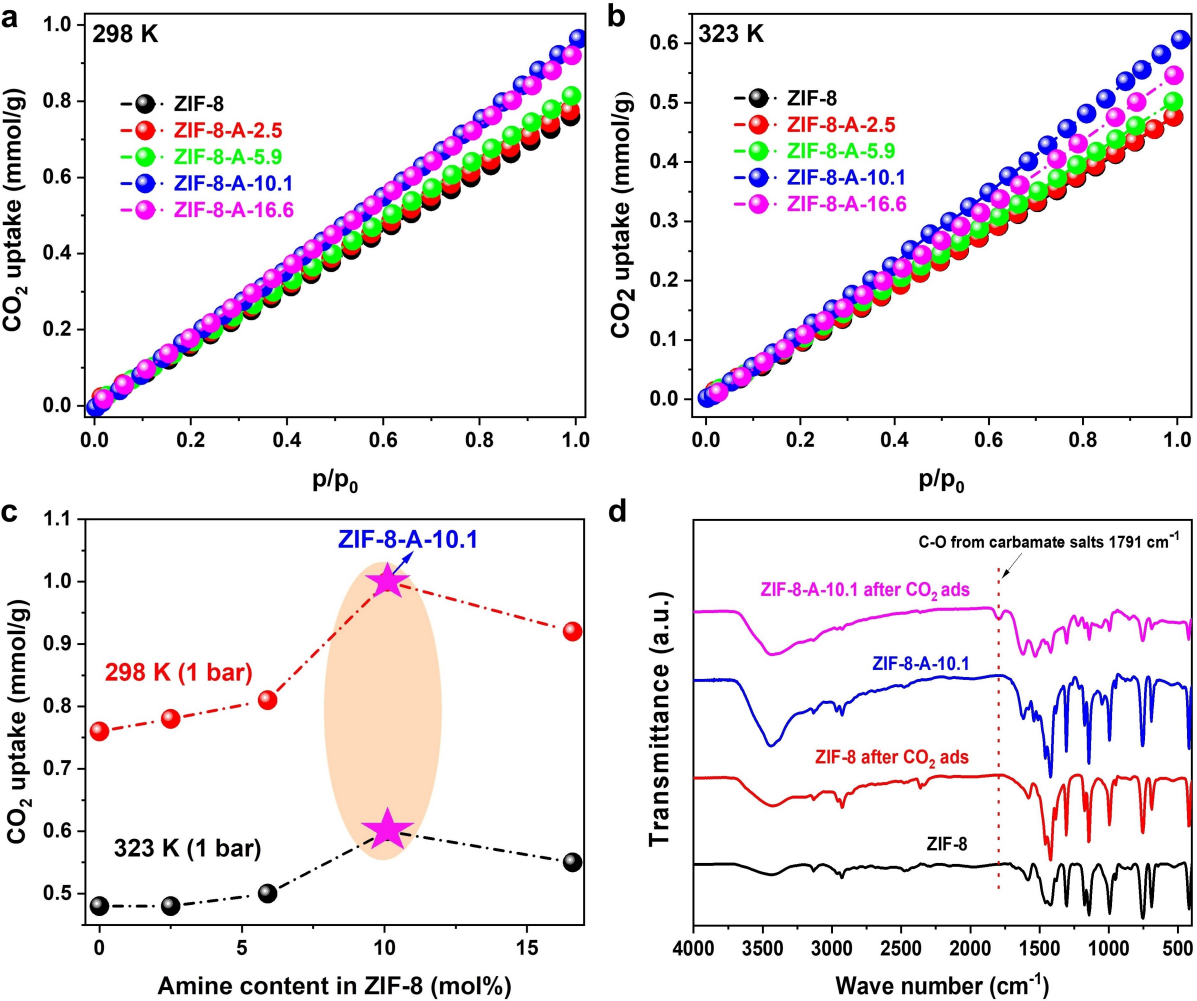
CO_2_ adsorption isotherm of microporous ZIF‐8 and amine functional & hierarchical mesoporous of ZIF‐8‐A materials at (a) 298 K, (b) 323 K, (c) The relationship between CO_2_ uptake amount with linker A contents in amine functional & hierarchical mesoporous ZIF‐8‐A, and (d) FT‐IR spectra of ZIF‐8 and ZIF‐8 A‐10.1 before and after CO_2_ adsorption.

The influence of amine‐functionalization within hierarchical mesoporous ZIF‐8‐A frameworks on CO_2_ adsorption was systematically studied. Data detailing CO_2_ adsorption capacities at two distinct temperatures (298 and 323 K) are delineated in Figure 5 and Table S4. At 1 bar and 298 K, ZIF‐8 demonstrated a CO_2_ adsorption capacity of 0.76 mmol g^−1^, aligning with findings from a previously published study.^[10]^ Significantly, an enhancement in CO_2_ uptake was observed with the incorporation of amine functionalities (A) into ZIF‐8‐A, which further augmented with an increase in the amine content at both 298 and 323 K. The sequence of CO_2_ adsorption capacities ascended as follows: ZIF‐8 < ZIF‐8‐A‐2.5 < ZIF‐8‐A‐5.9 < ZIF‐8–16.6 < ZIF‐8–10.1. This progression is attributable to the integration of free amine groups and a multitude of heterocyclic nitrogen‐containing pentagonal rings through the incorporation of amine A linker in ZIF‐8‐A. The presence of additional free amine sites and polar nitrogen electron‐rich groups within the triazole rings amplifies CO_2_ affinity through acid‐base interactions and N‐heterocyclic carbene (NHC)‐CO_2_ adduct formation.^[58]^ Moreover, Figure 5c illustrates a clear correlation between the concentration of amine linker A in ZIF‐8‐A and the respective CO_2_ uptake at 1 bar, across temperatures of 298 and 323 K. Intriguingly, ZIF‐8‐A‐10.1 exhibited better CO_2_ uptake compared to ZIF‐8‐A‐16.6, despite possessing a lower amine content. This phenomenon can be attributed to the larger hierarchical mesoporous size and greater density found in ZIF‐8‐A‐10.1, which enhances mass diffusion and subsequently the CO_2_ adsorption capacity within the mesoporous structure.

To investigate the CO_2_ adsorption mechanism on the materials, FT‐IR spectra were analyzed for ZIF‐8 and ZIF‐8‐A‐10.1 before and after CO_2_ adsorption (Figure 5d). The peak at 1791 cm^−1^ is associated with the formation of carbamate (or ‐NH_2_^+^‐CO_2_^−^ zwitterions),^[59]^ which occurs through the interaction between the adsorbed CO_2_ and electron‐rich amine groups present on the surface of ZIF‐8 A‐10.1, while this peak was not observed in ZIF‐8.

### The Effect of Functional Groups & Hierarchical Porous ZIF‐8 on Iodine Uptake

**Figure 6:**
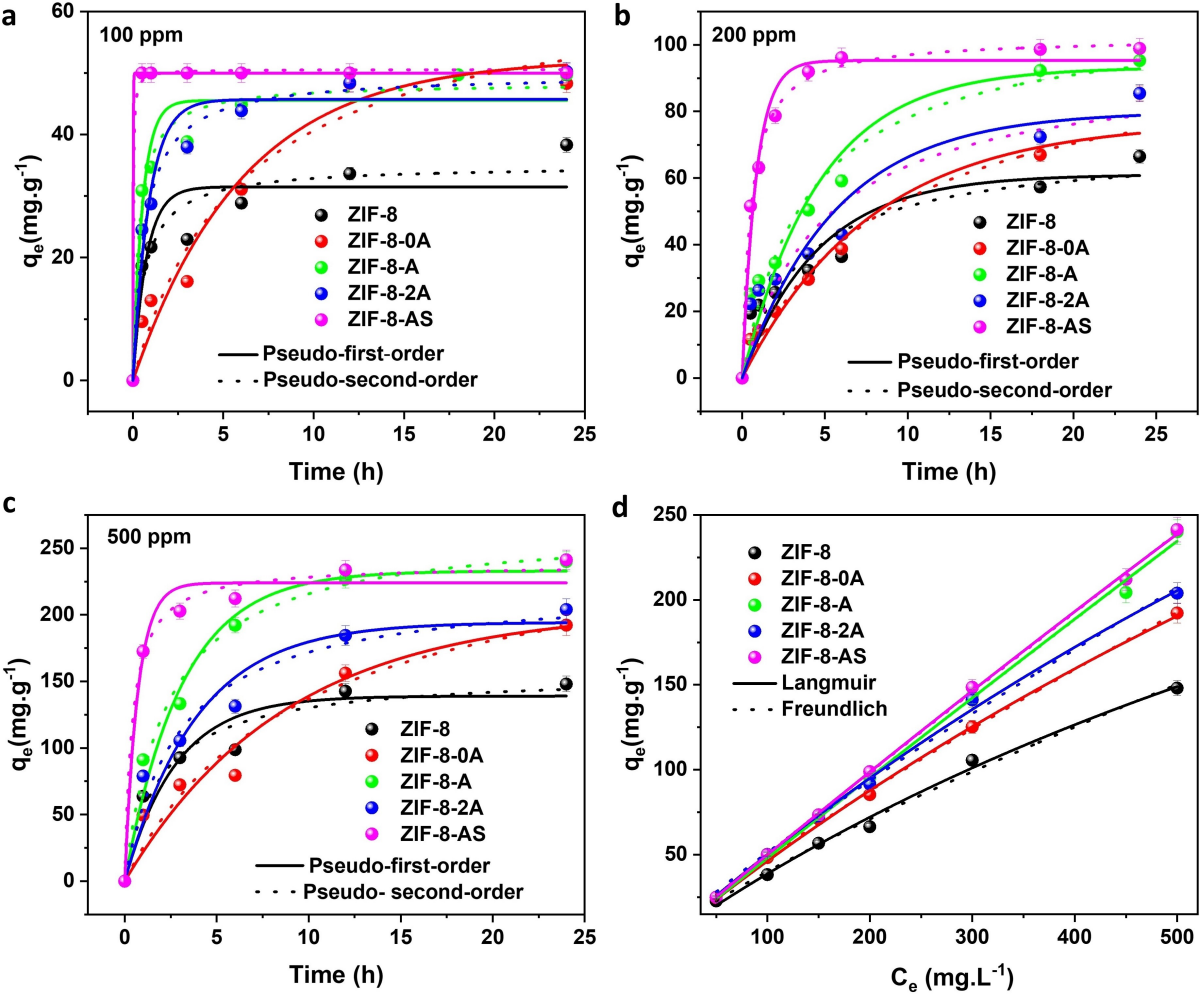
Kinetic adsorption of ZIF‐8 and various functional & hierarchical mesoporous ZIF‐8‐II materials, with corresponding Pseudo‐first‐order (PFO) and Pseudo‐second‐order (PSO) fitting at (a) 100 ppm, (b) 200 ppm, and (c) 500 ppm I_2_ in cyclohexane (absorbent: 2 mg mL^−1^, 24 hours, room temperature). (d) Adsorption Isotherms corresponding to the Langmuir and Freundlich fitting models for ZIF‐8 and ZIF‐8‐II materials over the I_2_ concentration range of 50–500 ppm.

The adsorption of iodine within ZIF‐8 materials was significantly influenced by several factors, including the presence of functional groups, surface area, pore volume, and the architecture of hierarchical mesoporous structures. The introduction of functional groups such as thiol (−SH) and amine (−NH_2_), alongside the electron‐rich heterocyclic triazole ring in ZIF‐8‐II, improved its interaction with iodine, given the role of iodine as an electron acceptor. The impact of these functional groups on the kinetics of iodine adsorption can be identified in Figures 6a–c, Figures S19–S21) at various iodine concentrations (100 ppm, 200 ppm, and 500 ppm). ZIF‐AS exhibited the fastest and highest adsorption kinetics among the ZIF‐8‐II series, likely due to strong chemical interactions between the thiol and amine groups with iodine, facilitated by Lewis acid‐base interactions and hydrogen bonding.

Notably, ZIF‐8‐A demonstrated superior adsorption kinetics than ZIF‐8–2 A, having two free amine groups. Theoretically, it could be enhanced to iodine adsorption by the improved hydrogen bonding and Lewis acid‐base interactions. This inconsistency can be described to the higher density of mesoporous regions in ZIF‐8‐A, enabling more favorable physical adsorption via the pore filling effect. Initially, ZIF‐8–0 A exhibits slower iodine adsorption kinetics compared to ZIF‐8 due to its smaller pore size, which restricts iodine diffusion. However, as iodine concentrates higher along the time within the pores, ZIF‐8–0 A exhibited enhanced adsorption capacity and kinetics, attributed to the conjugation of iodine with the triazole ring and strengthened Lewis acid‐base interactions. Additionally, the hydrogen bonding and I…π interactions between the triazole ring and iodine contributed to the observed increase in adsorption kinetics at later stages.

Among the functionalized ZIF‐8‐II variants, ZIF‐8–2 A outperforms ZIF‐8–0 A and ZIF‐8 in adsorption kinetics, likely due to the presence of two free amine groups that enhance interaction with iodine. Conversely, ZIF‐8–0 A exhibits the lowest kinetics among its counterparts, likely due to the absence of free functional groups and hierarchical mesoporosity. The order of iodine kinetic adsorption efficiency in functional and hierarchically porous ZIF‐8‐II materials is as follows: ZIF‐8 < ZIF‐8–0 A < ZIF‐8–2 A < ZIF‐8‐A < ZIF‐8‐AS, highlighting the effectiveness of functional groups for rapid iodine adsorption. In summary, the role of functional groups as electron‐donor entities and the incorporation of electron‐rich heterocyclic nitrogen centers from the triazole ring in ZIF‐8‐II significantly influence the kinetics of iodine adsorption. This influence was observed in the descending order of effectiveness: thiol groups in ZIF‐8‐AS, amine groups in ZIF‐8‐A, dual amine groups in ZIF‐8–2 A, the triazole ring in ZIF‐8–0 A, and finally, the imidazole ring in ZIF‐8.

### Kinetic Adsorption

The kinetic iodine adsorption on ZIF‐8 and ZIF‐8‐II materials was analyzed to understand the mechanism, with the models calculated using Equations (S3) and (S4) at various iodine concentrations (100 ppm, 200 ppm, and 500 ppm). As shown in Figure 6a–c, Table S5–S7, the pseudo‐second‐order kinetic model for ZIF‐8 and ZIF‐8‐II demonstrates a higher correlation coefficient (R^2^) than the pseudo‐first‐order model. Furthermore, the q_e_ values calculated from the pseudo‐second‐order model are more closely aligned with the experimental data than those calculated with the pseudo‐first‐order model (Tables S5–S7), suggesting that the interaction mechanism between iodine and materials is primarily driven by chemisorption.[^3^, ^4^]

### Adsorption Isotherms

To evaluate the iodine adsorption isotherms on the adsorbents, both the Langmuir and Freundlich isotherm models were fitted to the experimental data using Equations (S5) and (S6) to determine whether the adsorption occurs as a monolayer or involves multilayer adsorption.[^3^, ^4^] The findings reveal that the Langmuir model yields higher correlation coefficients (R^2^) than the Freundlich model (Figure 6d, Table 3) for iodine adsorption on ZIF‐8 and ZIF‐8‐II materials, suggesting that iodine adsorption on the heterogeneous surfaces of ZIF‐8 and ZIF‐8‐II occurs primarily as a monolayer. The maximum iodine adsorption capacities (q_m_) for ZIF‐8 were estimated to be around 514.3 mg g^−1^, while ZIF‐8‐A and ZIF‐8‐AS demonstrated significantly enhanced capacities with q_m_ values of approximately 1050.0 and 1101.5 mg g^−1^, respectively, nearly double compared to ZIF‐8. Table S8 summarizes the relationship between BET surface area, pore size, functional groups, and iodine adsorption for different ZIF‐8 materials. Although ZIF‐8 has the highest surface area and pore volume, it only contains 2 N‐ groups and lacks a hierarchical mesoporous structure, resulting in the lowest maximum iodine adsorption capacity (514.3 mg g^−1^) among the materials studied. In contrast, the ZIF‐8‐II series is significantly more effective at capturing I_2_ than pristine ZIF‐8 despite having a lower surface area and pore volume. This higher iodine adsorption capacity is attributed to the presence of various functional groups (−N, ‐NH₂, ‐SH) and a mesoporous structure. ZIF‐8‐AS exhibited the most rapid and highest adsorption kinetics among the ZIF‐8‐II series, which is attributed to the strong chemical interactions between thiol and amine groups with iodine, facilitated by Lewis acid‐base interactions and hydrogen bonding, despite having the lowest surface area and pore volume. This finding is consistent with previous research,[^5^, ^60^] which demonstrated that thiol functionalization in MIL‐53(Al) leads to higher iodine uptake (0.33 g g^−1^) compared to amine‐functionalized MIL‐53(NH_2_)(Al), which has an adsorption capacity of 0.18 g g^−1^. The maximum adsorption capacities increase in the following order, depending on the functional groups in the ZIF‐8‐II structure: ZIF‐8 < ZIF‐8–0 A < ZIF‐8–2 A < ZIF‐8‐A < ZIF‐8‐AS. This improvement in adsorption capacity indicates that the functional groups in the II ligand within the ZIF‐8‐II structure are crucial for I₂ adsorption. Similar to Falaise's finding,^[11]^ which investigated different functional groups (−OH,‐NH_2_) on various MOFs (MIL‐120, MIL‐101‐NH_2,_ MIL‐100, MIL53‐NH_2_, CAU‐1) for iodine adsorption, it was observed that the iodine absorption capacity of MOFs does not necessarily correlate with the surface area. Instead, they emphasized that electron donor groups, such as –NH_2_ and –OH, are crucial for iodine adsorption through charge transfer complexes between electron donor groups and iodine.

**Table 3:** Adsorption equilibrium constants of Langmuir and Freundlich isotherm models for I_2_ adsorption over ZIF‐8 and ZIF‐8‐II materials.

Material	Langmuir isotherm			Freundlich isotherm		
	q_max_ (mg. g^−1^)	K_L_ (L.mg^−1^)	R^2^	1/n	K_F_ (mg.mg^−1^)	R^2^
ZIF‐8	514.3	8.15*10^−4^	0.993	0.820	0.919	0.991
ZIF‐8–0 A	862.5	5.67*10^−4^	0.999	0.860	0.920	0.999
ZIF‐8‐A	1050.0	0.57*10^−4^	0.997	0.979	0.535	0.997
ZIF‐8–2 A	908.8	5.84*10^−4^	0.997	0.868	0.943	0.995
ZIF‐8‐AS	1101.5	1.14*10^−4^	0.999	0.969	0.581	0.999

Additionally, the maximum adsorption capacity of ZIF‐8‐A and ZIF‐8‐AS are highly competitive compared to other reported MOF absorbents (Figure S22 and Table S9). The high iodine adsorption performance of ZIF‐8‐A and ZIF‐8‐AS is attributed to the combined effect of their hierarchical porous structure and multiple active sites (amine/thiol groups, N and S atoms, and imidazole and triazole rings) present within the ZIF‐8‐A and ZIF‐8‐AS structures.

### Iodine Adsorption Mechanism

**Figure 7:**
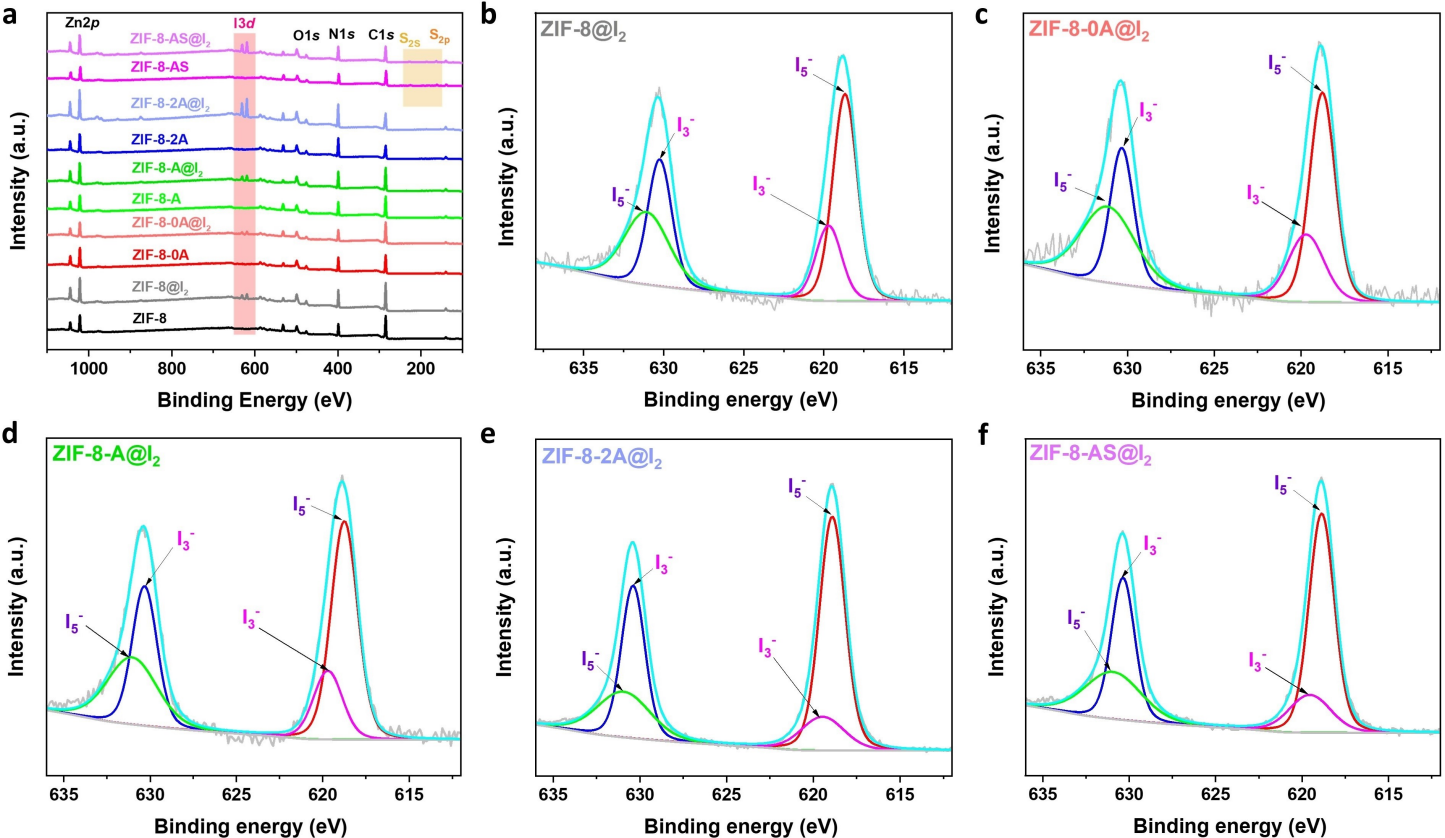
XPS spectra of ZIF‐8 and ZIF‐8‐II material before and after iodine adsorption, (a) Survey spectra, and narrow spectra of (b) ZIF‐8@I_2_, (c) ZIF‐8–0 A@I_2_, (d) ZIF‐8‐A@I_2_, (e) ZIF‐8–2 A@I_2_, and (f) ZIF‐8‐AS@I_2_.

To investigate the I₂ adsorption mechanism in ZIF‐8 and ZIF‐8‐II materials, X‐ray photoelectron spectroscopy (XPS) and Raman spectroscopy analyses were conducted on these materials before and after I₂ adsorption (Figures 7, S23, and S24). The spectra of ZIF‐8@I₂ and ZIF‐8‐II@I₂ (Figure 7) display two main peaks corresponding to I₃^−^ (~630.3 and 619.7 eV) and I₅^−^ (~631.1 and 618.8 eV).[^4^, ^61^] These observations indicate that the adsorbed I₂ molecules on ZIF‐8 and ZIF‐8‐II were converted into I₃^−^ and I₅^−^ due to chemical interactions with the ZIF‐8 and ZIF‐8‐II structures.^[3]^ Furthermore, the N1s narrow peak in ZIF‐8@I₂ shows a similar profile to that of pristine ZIF‐8, while the N 1s and S 2p peaks in ZIF‐8‐II@I₂ are slightly shifted to higher binding energies compared to those in ZIF‐8‐II (Figure S23). This suggests a reduction in electron density around the N and S atoms after I₂ adsorption, likely due to electron transfer from the N and S atoms in the imidazole and II ligands to the iodine atoms in ZIF‐8‐II@I₂.^[3]^ These results confirm the strong chemical interaction between N‐functionalized groups/S‐groups and iodine species.

The Raman spectra of ZIF‐8@I₂ and ZIF‐8‐II@I₂ (Figure S24) reveal three additional peaks that correspond to polyiodine anions (I₃^−^, I₅^−^),[^11^, ^61^] when compared to the pristine ZIF‐8 and ZIF‐8‐II. These findings indicate that the chemical interaction between the ZIF‐8 and ZIF‐8‐II materials and adsorbed I₂ occurs via charge transfer from the nitrogen electron donors to the σ* orbital of I₂, which then transforms into I₃^−^ or I₅^−^ within the ZIF‐8 and ZIF‐8‐II frameworks, consistent with the XPS data presented above.[^3^, ^61^, ^62^, ^63^, ^64^] Interestingly, ZIF‐8‐II@I₂ spectrum displays relatively strong polyiodine anion bands with higher intensity compared to ZIF‐8@I₂, indicating a stronger chemical interaction between ZIF‐8‐II and I₂ (involving N…I, NH⋅⋅⋅I, H₂N⋅⋅⋅I, and HS…I interactions). The notably higher intensity of ZIF‐8‐II@I₂ compared to ZIF‐8@I₂ suggests a stronger interaction between the functional groups in ZIF‐8‐II and iodine than in ZIF‐8.

**Figure 8:**
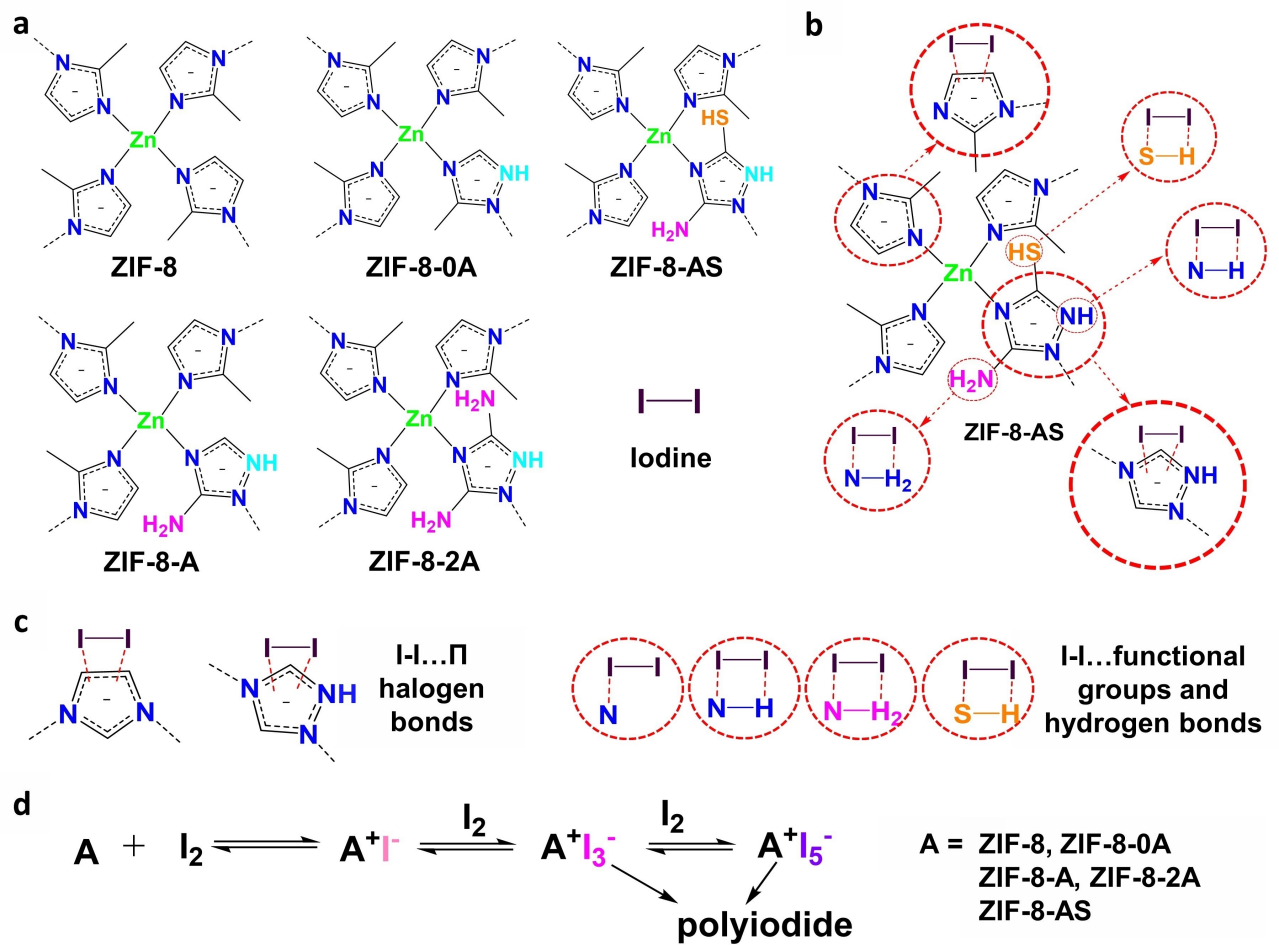
(a) Structure of adsorbents ZIF‐8, ZIF‐8‐II, and iodine, (b) Scheme interaction between ZIF‐8‐AS and iodine, (c) Possible interactions between iodine and rich electron donor ring (imidazole and triazole ring) through halogen bonds interaction and electron transfer, functional groups (−N, ‐NH,‐NH_2_ and ‐SH) through electron transfer and hydrogen bonding, (d) Schematic formation of polyiodide between adsorbents (A:ZIF‐8/ZIF‐8‐II) and iodine.

Finally, combining the experimental results from Raman and XPS analysis with previous reports,[^3^, ^5^, ^6^, ^11^, ^62^] the plausible iodine adsorption mechanism is illustrated in Figure 8. Figure 8a–c displays the structures of ZIF‐8 and ZIF‐8‐II materials, the interaction between ZIF‐8‐AS and iodine, and the interaction between ZIF‐8, ZIF‐8‐II, and iodine. These interactions occur through electron transfer involving I−I…π (halogen bonds) between iodine and electron‐rich heteroatoms in the imidazole/triazole rings, I−I…functional groups (−N,‐NH,‐NH_2_ and ‐SH), and hydrogen bonds. Additionally, it presents a possible scheme for the formation of polyiodides (AI_3_^−^ and AI_5_^−^) between the materials and I₂ (Figure 8d). Specifically, ZIF‐8‐II series and ZIF‐8, which incorporate free thiol (−SH), free amine (−NH_2_) groups and nitrogen‐containing electron‐rich triazole/ imidazole rings, offered numerous sites for iodine adsorption. These sites acts as Lewis bases, interacting with the Lewis acidic (iodine molecules) through charge transfer from the electron‐donating species to σ* orbital of I_2_, leading to the formation of a charge‐transfer complex and subsequently absorb additional iodine molecules, resulting in the formation of polyiodide complexes such as AI_3_^−^ and AI_5_^−^ (Figure 8d).^[62]^ Especially, ZIF‐8‐AS was distinguished by its thiol groups (−SH), which engage in interactions with iodine to form covalent sulfenyl iodide (S−I) bond through Lewis acid‐base interactions, supplemented by hydrogen bonding and pore filling.[^60^, ^65^] ZIF‐8–0 A engaged in coordination between its amine groups and iodine, facilitated by Lewis acid‐base interactions and hydrogen bonding through NH…I and N…I bonding. The I…π halogen bond interaction between the triazole ring and iodinated bonds through electron transfer, resulting in the formation of I_3_^−^, I_5_^−^ and pore filling.[^66^, ^67^]

### Recyclability, and Reusability Study

The reusability of the adsorbents was evaluated over three cycles, using ethanol as desorption reagent. As shown in Figure S25, ZIF‐8 retained the I_2_ removal efficiency after reuse, while ZIF‐8‐II exhibited a slight decrease in efficiency. This reduction is likely due to some I_2_ molecules remaining trapped within the functional groups and mesoporousity of ZIF‐8‐II materials, caused by strong interactions between the functional groups in ZIF‐8‐II with iodine molecules.^[3]^ The XRD pattern and FTIR analysis confirm that the ZIF‐8 and ZIF‐8‐II materials maintain their crystallinity and structural integrity after multiple cycles, demonstrating their durability and reusability.

## Conclusions

In summary, amine and thiol‐functionalized hierarchical mesoporous ZIF‐8 were successfully synthesized using *de sono* mixed ligand synthesis strategy aimed at CO_2_ and iodine adsorption applications. This research outlines a detailed method for manipulating particle size, hierarchical mesoporous structures, and functional groups within ZIF‐8 through a mixed multivariate linker strategy. The incorporation of dual ligands with amine and thiol functional groups, combined with heterocyclic nitrogen‐containing five‐membered rings, enhances the synergy effect. This effect offers multiple active sites for iodine and CO_2_ chemisorption and improves mass diffusion. The synthesized hierarchical mesoporous ZIF‐8, enriched with various functional groups, shows a significant increase in CO_2_ and iodine adsorption capacities. Among the materials, ZIF‐8‐AS, with amine and thiol functional groups, demonstrates the fastest adsorption kinetics and achieves a twofold increase in iodine adsorption capacity (q_m_=1101.5 mg g^−1^) compared to ZIF‐8. Furthermore, the hierarchical porous structure of amine‐functionalized ZIF‐8‐A significantly boosts CO_2_ uptake, with ZIF‐8‐A‐10.1, which has the highest degree of mesoporosity, achieving a CO_2_ adsorption capacity of 1.0 mmol g^−1^ at 298 K, which is 1.3 times higher than that of microporous ZIF‐8. The straightforward and room‐temperature synthesis process underscores its potential for industrial applications, especially considering its scalability for mass production. The methodology presented here offers a scalable and effective way to design ZIF‐8 with desired functionalities and hierarchical mesoporosity for diverse applications, marking a step forward in adsorption technology. This study introduces an eco‐friendly and highly efficient route to synthesize functionally diverse and hierarchically porous ZIF‐8.

## Conflict of Interests

The authors declare no competing financial interest.

## Supporting information

As a service to our authors and readers, this journal provides supporting information supplied by the authors. Such materials are peer reviewed and may be re‐organized for online delivery, but are not copy‐edited or typeset. Technical support issues arising from supporting information (other than missing files) should be addressed to the authors.

Supporting Information

## Data Availability

Research data are not shared.

## References

[1] P. Tyagi , D. Singh , N. Malik , S. Kumar , R. Singh Malik , Mater. Today. 2023, 65, 133–165.

[2] T. K. Pal , D. De , P. K. Bharadwaj , Coord. Chem. Rev. 2020, 408, 213173.

[3] K. Y. Baek , Y. R. Lee , X. H. Do , K. Y. Cho , K. Jeong , ACS Appl. Nano Mater. 2020, 3, 9852–9861.

[4] Z. W. Wang , K. W. Chen , A. T. Gu , X. Y. Zhou , P. Wang , C. H. Gong , P. Mao , Y. Jiao , K. Chen , J. G. Lu , Y. Yang , J. Solid State Chem. 2023, 325, 124186.

[5] X. Zhang , J. Maddock , T. M. Nenoff , M. A. Denecke , S. Yang , M. Schröder , Chem. Soc. Rev. 2022, 51, 3243–3262. 35363235 10.1039/d0cs01192d PMC9328120

[6] T. Pan , K. Yang , X. Dong , Y. Han , J. Mater. Chem. A. 2023, 11, 5460–5475.

[7] J. López-Cabrelles , E. Miguel-Casañ , M. Esteve-Rochina , E. Andres-Garcia , I. J. Vitórica-Yrezábal , J. Calbo , G. Mínguez Espallargas , Chem. Sci. 2022, 13, 842–847. 35173949 10.1039/d1sc04779e PMC8768878

[8] H. Furukawa , K. E. Cordova , M. O'Keeffe , O. M. Yaghi , Science 2013, 341, 1230444. 23990564 10.1126/science.1230444

[9] A. Akhundzadeh Tezerjani , R. Halladj , S. Askari , RSC Adv. 2021, 11, 19914–19923. 35479238 10.1039/d1ra02856a PMC9033796

[10] S. Gadipelli , W. Travis , W. Zhou , Z. Guo , Energy Environ. Sci. 2014, 7, 2232–2238.

[11] C. Falaise , C. Volkringer , J. Facqueur , T. Bousquet , L. Gasnot , T. Loiseau , Chem. Commun. 2013, 49, 10320–10322. 10.1039/c3cc43728k 24067882

[12] D. F. Sava , M. A. Rodriguez , K. W. Chapman , P. J. Chupas , J. A. Greathouse , P. S. Crozier , T. M. Nenoff , J. Am. Chem. Soc. 2011, 133, 12398–12401. 21766858 10.1021/ja204757x

[13] K. Y. Cho , H. An , X. H. Do , K. Choi , H. G. Yoon , H. K. Jeong , J. S. Lee , K. Y. Baek , J. Mater. Chem. A. 2018, 6, 18912–18919.

[14] D. Yu , Q. Shao , Q. Song , J. Cui , Y. Zhang , B. Wu , L. Ge , Y. Wang , Y. Zhang , Y. Qin , R. Vajtai , P. M. Ajayan , H. Wang , T. Xu , Y. Wu , Nat. Commun. 2020, 11, 927. 32066754 10.1038/s41467-020-14671-9 PMC7026438

[15] M. Sadakiyo , T. Kuramoto , K. Kato , M. Yamauchi , Chem. Lett. 2017, 46, 1004–1006.

[16] O. Karagiaridi , M. B. Lalonde , W. Bury , A. A. Sarjeant , O. K. Farha , J. T. Hupp , J. Am. Chem. Soc. 2012, 134, 18790–18796. 23088345 10.1021/ja308786r

[17] Y. W. Abraha , C. W. Tsai , J. W. H. Niemantsverdriet , E. H. G. Langner , ACS Omega 2021, 6, 21850–21860. 34497880 10.1021/acsomega.1c01130 PMC8412924

[18] C. Tsai , J. W. Niemantsverdriet , E. H. G. Langner , Microporous Mesoporous Mater. 2018, 262, 98–105.

[19] Z. Jiang , W. Xue , H. Huang , H. Zhu , Y. Sun , C. Zhong , Chem. Eng. J. 2023, 454, 140093.

[20] Y. V. Kaneti , S. Dutta , M. S. A. Hossain , M. J. A. Shiddiky , K. L. Tung , F. K. Shieh , C. K. Tsung , K. C. W. Wu , Y. Yamauchi , Adv. Mater. 2017, 29, 1700213. 10.1002/adma.201700213 28833624

[21] J. Q. Jiang , C. X. Yang , X. P. Yan , Chem. Commun. 2015, 51, 6540–6543. 10.1039/c5cc00366k 25767058

[22] K. C. Jayachandrababu , D. S. Sholl , S. Nair , J. Am. Chem. Soc. 2017, 139, 5906–5915. 28388071 10.1021/jacs.7b01660

[23] W. Wu , J. Su , M. Jia , Z. Li , G. Liu , W. Li , Sci. Adv. 2020, 6, eaax7270. 32494660 10.1126/sciadv.aax7270 PMC7195121

[24] J. P. Zhang , A. X. Zhu , R. B. Lin , X. L. Qi , X. M. Chen , Adv. Mater. 2011, 23, 1268–1271. 21381128 10.1002/adma.201004028

[25] K. Eum , K. C. Jayachandrababu , F. Rashidi , K. Zhang , J. Leisen , S. Graham , R. P. Lively , R. R. Chance , D. S. Sholl , C. W. Jones , S. Nair , J. Am. Chem. Soc. 2015, 137, 4191–4197. 25774460 10.1021/jacs.5b00803

[26] J. A. Thompson , N. A. Brunelli , R. P. Lively , J. R. Johnson , C. W. Jones , S. Nair , J. Phys. Chem. C. 2013, 117, 8198–8207.

[27] L. G. Qiu , T. Xu , Z. Q. Li , W. Wang , Y. Wu , X. Jiang , X. Y. Tian , L. De Zhang , Angew. Chem. Int. Ed. 2008, 47, 9487–9491. 10.1002/anie.200803640 18972472

[28] Y. N. Wu , M. Zhou , B. Zhang , B. Wu , J. Li , J. Qiao , X. Guan , F. Li , Nanoscale 2014, 6, 1105–1112. 24296611 10.1039/c3nr04390h

[29] K. Shen , L. Zhang , X. Chen , L. Liu , D. Zhang , Y. Han , J. Chen , J. Long , R. Luque , Y. Li , B. Chen , Science 2018, 359, 206–210. 29326271 10.1126/science.aao3403

[30] H. N. Abdelhamid , Z. Huang , A. M. El-Zohry , H. Zheng , X. Zou , Inorg. Chem. 2017, 56, 9139–9146. 28715176 10.1021/acs.inorgchem.7b01191

[31] J. Yang , F. Zhang , H. Lu , X. Hong , H. Jiang , Y. Wu , Y. Li , Angew. Chem. 2015, 127, 11039–11043.

[32] Z. Qin , H. Li , X. Yang , L. Chen , Y. Li , K. Shen , Appl. Catal. B Environ. 2022, 307, 121163.

[33] H. Li , F. Meng , S. Zhang , L. Wang , M. Li , L. Ma , W. Zhang , W. Zhang , Z. Yang , T. Wu , S. Lee , F. Huo , J. Lu , Angew.Chem. Int. Ed. 2020, 59, 2457–2464. 10.1002/anie.201912972 31769126

[34] L. Y. Chou , P. Hu , J. Zhuang , J. V. Morabito , K. C. Ng , Y. C. Kao , S. C. Wang , F. K. Shieh , C. H. Kuo , C. K. Tsung , Nanoscale. 2015, 7, 19408–19412. 26538214 10.1039/c5nr06532a

[35] H. J. Lee , W. Cho , M. Oh , Chem. Commun. 2012, 48, 221–223. 10.1039/c1cc16213f 22089881

[36] Y. Chuan Tan , H. Chun Zeng , Chem. Commun. 2016, 52, 11591–11594. 10.1039/c6cc05699g 27604286

[37] Z. Huang , J. Rath , Q. Zhou , A. Cherevan , S. Naghdi , D. Eder , Small 2023, 20, 2307981. 38126913 10.1002/smll.202307981 PMC11478943

[38] K. Zhou , B. Mousavi , Z. Luo , S. Phatanasri , S. Chaemchuen , F. Verpoort , J. Mater. Chem. A. 2017, 5, 952–957.

[39] Q. T. Nguyen , X. H. Do , K. Y. Cho , Y. R. Lee , K. Y. Baek , J. CO2 Util. 2022, 61, 102061.

[40] K. S. Park , Z. Ni , A. P. Côté , J. Y. Choi , R. Huang , F. J. Uribe-Romo , H. K. Chae , M. O'Keeffe , O. M. Yaghi , Proc. Natl. Acad. Sci. U. S. A 2006, 103, 10186–10191. 16798880 10.1073/pnas.0602439103 PMC1502432

[41] D. Villalgordo-Hernández , M. Antonio Diaz-Perez , V. Balloi , M. Anabel Lara-Angulo , J. Narciso , J. Carlos Serrano-Ruiz , E. V. Ramos-Fernandez , Chem. Eng. J. 2023, 476, 146846. 10.3390/molecules28166095 PMC10458508 37630347

[42] H. An , K. Y. Cho , Q. Lyu , D. S. Chiou , K. J. Nam , D. Y. Kang , L. C. Lin , J. S. Lee , Adv. Funct. Mater. 2021, 31, 2105577.

[43] H. T. Kwon , H. K. Jeong , A. S. Lee , H. S. An , J. S. Lee , J. Am. Chem. Soc. 2015, 137, 12304–12311. 26364888 10.1021/jacs.5b06730

[44] C. Healy , K. M. Patil , B. H. Wilson , L. Hermanspahn , N. C. Harvey-Reid , B. I. Howard , C. Kleinjan , J. Kolien , F. Payet , S. G. Telfer , P. E. Kruger , T. D. Bennett , Coord. Chem. Rev. 2020, 419, 213388.

[45] H. Deng , C. J. Doonan , H. Furukawa , R. B. Ferreira , J. Towne , C. B. Knobler , B. Wang , O. M. Yaghi , Science 2010, 327, 846–850. 20150497 10.1126/science.1181761

[46] M. Kandiah , M. H. Nilsen , S. Usseglio , S. Jakobsen , U. Olsbye , M. Tilset , C. Larabi , E. A. Quadrelli , F. Bonino , K. P. Lillerud , Chem. Mater. 2010, 22, 6632–6640.

[47] M. Loloei , S. Kaliaguine , D. Rodrigue , Ind. Eng. Chem. Res. 2022, 61, 10.

[48] M. Erkartal , U. Erkilic , B. Tam , H. Usta , O. Yazaydin , J. T. Hupp , O. K. Farha , U. Sen , Chem. Commun. 2017, 53, 2028–2031. 10.1039/c6cc08746a 28124040

[49] J. Cravillon , R. Nayuk , S. Springer , A. Feldhoff , K. Huber , M. Wiebcke , Chem. Mater. 2011, 23, 2130–2141.

[50] P. Deria , J. E. Mondloch , O. Karagiaridi , W. Bury , J. T. Hupp , O. K. Farha , Chem. Soc. Rev. 2014, 43, 5896–5912. 24723093 10.1039/c4cs00067f

[51] V. Colombo , S. Galli , H. J. Choi , G. D. Han , A. Maspero , G. Palmisano , N. Masciocchi , J. R. Long , Chem. Sci. 2011, 2, 1311–1319.

[52] S. Bhattacharyya , R. Han , W. G. Kim , Y. Chiang , K. C. Jayachandrababu , J. T. Hungerford , M. R. Dutzer , C. Ma , K. S. Walton , D. S. Sholl , S. Nair , Chem. Mater. 2018, 30, 4089–4101.

[53] J. Di Lin , F. Chen , J. G. Xu , F. K. Zheng , N. Wen , ACS Appl. Nano Mater. 2019, 2, 5116–5124.

[54] Z. Shi , Y. Tao , J. Wu , C. Zhang , H. He , L. Long , Y. Lee , J. Am. Chem. Soc. 2020, 142, 2750–2754. 31968944 10.1021/jacs.9b12879

[55] X. Xu , Y. Sun , Q. Zhang , S. Wang , L. Zhang , Z. Wu , G. Lu , ChemistrySelect. 2016, 1, 1763–1767.

[56] S. He , Y. Chen , Z. Zhang , B. Ni , W. He , X. Wang , Chem. Sci. 2016, 7, 7101–7105. 28567265 10.1039/c6sc02272c PMC5450591

[57] C. Duan , F. Li , J. Xiao , Z. Liu , C. Li , H. Xi , Sci. China Mater. 2017, 60, 1205–1214.

[58] X. Wang , Q. Dong , Z. Xu , Y. Wu , D. Gao , Y. Xu , C. Ye , Y. Wen , A. Liu , Z. Long , G. Chen , Chem. Eng. J. 2021, 403, 126460.

[59] Q. Thi Nguyen , Y. Bae , X. H. Do , S. H. Kim , J. Na , K. Y. Baek , Y. R. Lee , Energy Fuels 2024, 38, 7097–7107.

[60] A. S. Munn , F. Millange , M. Frigoli , N. Guillou , C. Falaise , V. Stevenson , C. Volkringer , T. Loiseau , G. Cibin , R. I. Walton , CrystEngComm. 2016, 18, 8108–8114.

[61] W. Zhou , A. Li , M. Zhou , Y. Xu , Y. Zhang , Q. He , Nat. Commun. 2023, 14, 5388. 37666841 10.1038/s41467-023-41056-5 PMC10477329

[62] J. Wang , T. Wu , X. Wang , J. Chen , M. Fan , Z. Shi , J. Liu , L. Xu , Y. Zang , iScience 2024, 27, 108993. 38327786 10.1016/j.isci.2024.108993 PMC10847683

[63] L. Chen , J. Y. Qian , D. D. Zhu , S. Yang , J. Lin , M. Y. He , Z. H. Zhang , Q. Chen , ACS Appl. Nano Mater. 2020, 3, 5390–5398.

[64] P. Chen , X. He , M. Pang , X. Dong , S. Zhao , W. Zhang , ACS Appl. Mater. Interfaces 2020, 12, 20429–20439. 32255599 10.1021/acsami.0c02129

[65] K. K. Yee , Y. L. Wong , Z. Xu , Dalt. Trans. 2016, 45, 5334–5338. 10.1039/c6dt00016a 26902735

[66] T. Xu , J. Li , M. Jia , G. Li , Y. Liu , Dalt. Trans. 2021, 50, 13096–13102. 10.1039/d1dt00947h 34581332

[67] J. Qin , W. Zhang , Y. Chen , R. Liu , Y. Fan , Environ. Sci. Pollut. Res. 2021, 28, 28797–28807. 10.1007/s11356-021-12588-4 33548041

