# Supplementary material for: Multifunctional and Hierarchical Porous ZIF‐8: Amine and Thiol Tagged via Mixed Multivariate Ligand Strategies for Enhanced CO2 and Iodine Adsorption
Source: ChemSusChem. 2024 Nov 7;18(4):e202401968. doi: 10.1002/cssc.202401968 (PMC13066728; doi:10.1002/cssc.202401968)
Supplement: Supplementary file 1 — Supporting Information [file CSSC-18-e202401968-s001.pdf]

ChemSusChem

Supporting Information

Multifunctional and Hierarchical Porous ZIF-8: Amine and Thiol Tagged via Mixed Multivariate Ligand Strategies for Enhanced CO_2 and Iodine Adsorption

Que Thi Nguyen, Jun Young Lee, Yejin Bae, Yu-Ri Lee, Younghan Song, Sang Hoon Kim,* Kyung-Youl Baek,* and Jongbeom Na*

Supporting Information

Multifunctional and Hierarchical Porous ZIF-8: Amine and Thiol Tagged via Mixed Multivariate Ligand Strategies for Enhanced CO_2 and Iodine Adsorption

Que Thi Nguyen,[a,b,c] Jun Young Lee,[a] Yejin Bae,[d,e] Yu-Ri Lee,[e] Younghan Song,[a] Sang Hoon Kim,[b,c,f]* Kyung-Youl Baek, [a,c]†* and Jongbeom Na[a,f,g]*

[a] Q.T. Nguyen, J. Y. Lee, Dr. Y. Song, Prof. Dr. K-Y. Baek, Dr. J. Na
Materials Architecturing Research Center, Korea Institute of Science and Technology, Seoul, 02792, Republic of Korea
E-mail: kim_sh@kist.re.kr; jongbeom@kist.re.kr
[b] Q.T. Nguyen, Prof. Dr. S. H. Kim
Extreme Materials Research Center, Korea Institute of Science and Technology, Seoul, 02792, Republic of Korea
[c] Q.T. Nguyen, Prof. Dr. S. H. Kim, Prof. Dr. K-Y. Baek
Division of Nanoscience and Technology, KIST School, University of Science and Technology, Daejeon 34113, Republic of Korea
[d] Y. Bae
Department of Energy Engineering, Hanyang University, Seoul 04763, Republic of Korea
[e] Y. Bae, Dr. Y-R. Lee
Greenhouse Gas Research Laboratory, Korea Institute of Energy Research, Daejeon 305-343, Republic of Korea
[f] Dr. S. H. Kim, Dr. J. Na
KHU-KIST Department of Converging Science and Technology, Kyung Hee University, Seoul 02447, Republic of Korea
[g] Dr. J. Na
Australian Institute for Bioengineering and Nanotechnology (AIBN), The University of Queensland, Brisbane, Queensland 4072, Australia

† *deceased 3rd October 2023.*

Number of pages: 40 (S1-S40)

Number of figures: 25 (Figure S1-S25)

Number of tables: 9 (Table S1-S9)

Experimental section

Chemicals and reagents

Zinc nitrate hexahydrate ($Zn(NO_3)_2 \cdot 6H_2O$, 99%), 2-methylimidazole (Hmim, 99%), 1,2,4-triazole (0A, 99%), 3- amino-1,2,4-triazole (A, 99%), 3,5 diamino-1,2,4-triazole (2A, 99%), 3-amino-1,2,4-triazole-5-thiol (AS, 98%), Iodine (99.9%), cyclohexane (99.9%), and methanol (MeOH, 99.9%) were purchased from Sigma-Aldrich, Alfa-Aesar and Daejung. Chloroform-d ($CDCl_3$, 99.8%) and dimethyl sulfoxide-d6 (DMSO-d6, 99.9%) used as NMR solvents were obtained from Cambridge isotope laboratories. All chemicals were used without further purification.

Synthesis of ZIF-0A, ZIF-A, and ZIF-2A materials

ZIF-0A was synthesized using a procedure similar to the synthesis of microporous ZIF-8, utilizing $Zn(NO_3)_2 \cdot 6H_2O$ and 1,2,4-triazole (0A) as metal salts and ligand. Specifically, 0.48 g (1.6 mmol) of $Zn(NO_3)_2.6H_2O$ and 0.89 g (12.8 mmol) of linker 0A were dissolved separately in 32 mL and 16 mL of methanol, respectively, under stirring for 30 minutes at room temperature. The metal salt mixture was then added to the ligand mixture under stirring for 24 hours. The resulting white precipitate product was collected by centrifuge at 10000 rpm and washed with methanol for several times. Finally, ZIF-0A was dried in a vacuum at 100 ℃ for 24 hours.

ZIF-A, and ZIF-2A were synthesized using a similar procedure as ZIF-0A, with the only difference being the replacement of 1,2,4-triazole (0A) linker by 3- amino-1,2,4-triazole (A) and 3,5 diamino-1,2,4-triazole (2A) ligand, respectively.

Ligand exchange of ZIF-8 with different ligands (0A, A, 2A)

0.231 g (1 mmol) of ZIF-8 was dispersed well in 50 mL methanol and then 1.24 g (18 mmol) of 1,2,4-triazole (0A) was added to ZIF-8 suspension. The mixture was kept stirring at room temperature for 24 hours. After the reaction, the product was separated by centrifugation and washed with methanol several times, and dried at 100 ℃ on a vacuum overnight. The corresponding product was designated as ZIF-8-0AE. ZIF-8-AE and ZIF-8-2AE were synthesized using a similar procedure as ZIF-8-0A, with the only difference being the replacement of the 1,2,4-triazole (0A) linker in ligand exchange by 3-

amino-1,2,4-triazole (A) and 3,5-diamino-1,2,4-triazole (2A) ligands, respectively.

Ligand exchange of ZIF-0A, ZIF-A, ZIF-2A with 2-methyl imidazole ligand (Hmim)

0.205 g (1 mmol) of ZIF-0A was dispersed well in 50 mL methanol, and then 1.478 g (18 mmol) of Hmim was added to ZIF-0A suspension. The mixture was kept stirring at room temperature for 24 hours. After the reaction, the product was separated by centrifugation and washed with methanol several times, and dried at 100 ℃ on a vacuum overnight. The corresponding product was designated as ZIF-0A-8E. A similar procedure was followed for ligand exchange with ZIF-A and ZIF-2A, resulting in samples ZIF-A-8E and ZIF-2A-8E, respectively.

Iodine adsorption

*S*olution of iodine (I_2) with concentration of 500, 450, 400, 350, 300, 200, 150, 100, and 50 mg.L^{-1} in cyclohexane were prepared. Before I_2 adsorption, the ZIF-8 and functional hierarchical porous ZIF-8 adsorbents were pretreated at 100 °C under vacuum conditions for overnight. In a vial, 30 mL of iodine solution in cyclohexane was added with stirring. Subsequently, the absorbents were added with concentration 2 mg·mL^{-1}, followed by sonication for 5 minutes and further stirring at room temperature. At intervals, small samples were collected, and the I_2 solution was separated from adsorbents using a 0.22 μm polyethylene terephthalate (PET) syringe filter. The concentrations of I_2 were determined by measuring its ultraviolet (UV)-visible absorption at 523 nm and referring to a calibration curve. The I_2 adsorption capacities were calculated using the following equation S1:[1–4]

$$q_{e(t)} = \left(C_1 - C_{e(t)}\right) \times \frac{V}{m} \quad (S1)$$

The iodine removal efficiency (%) was calculated using equation S2:

$$Removal\ (\%) = \frac{\left(C_i - C_{e(t)}\right)}{C_i} \times 100\% \quad (S2)$$

where C_t and C_e represent for iodine concentration at time t and at equilibrium (mg.L^{-1}), C_i is the initial iodine concentration (mg·L^{-1}), V is the solution volume (L), and m is the mass of the adsorbent (g), q_e and q_t are the iodine adsorption capacities at time t and at equilibrium (mg·g^{-1}).

Reusability tests were conducted by immersing the materials (ZIF-8 and ZIF-8-II) in an I_2 solution (500

ppm) at room temperature for 24 hours with stirring to achieve saturation. After adsorption, the materials were filtered to remove iodine solution, then soaked and sonicated in ethanol, followed by another round filtration. This washing process was repeated several times. The materials were then dried under vacuum at 100 ℃ for 24 hours.

Adsorption Kinetics Study

To evaluate the kinetics of I_2 adsorption on ZIF-8 and ZIF-8-II materials, the pseudo-first-order (PFO) and pseudo-second-order (PSO) kinetic models were employed to fit the experimental data by equation (S3) and (S4), respectively:[1–3]

$$q_t = q_e(1 - e^{-k_1 t}) \ (S3)$$

$$q_t = \frac{k_2 {q_e}^2 t}{1 + k_2 q_e t} \quad (S4)$$

In these equations, q_e and q_t represent the amounts of I_2 adsorbed by the adsorbent ($mg{\cdot}g^{-1}$) at equilibrium and at a given time (t), respectively. The constants k_1 (h^{-1}) and k_2 (g $mg^{-1}{\cdot}h^{-1}$) represent the rate constants of the PFO and PSO models, respectively.

Adsorption Isotherms Study

The adsorption isotherms were analyzed using the Langmuir and Freundlich models. The Langmuir isotherm model, which assumes monolayer adsorption on a uniform surface, is expressed by equation (S5). In contrast, the Freundlich model, which assumes adsorption on a heterogeneous surface, is described by equation (S6).[1,2]

$$q_e = \frac{q_m K_L C_e}{1 + K_L C_e} \ (S5)$$

$$q_e = K_F C_e^{1/n} \ (S6)$$

In these equations, q_e ($mg{\cdot}g^{-1}$) is the amount of I_2 adsorbed per unit of adsorbent at equilibrium, q_m ($mg{\cdot}g^{-1}$) represents the maximum adsorption capacity, and C_e ($mg{\cdot}L^{-1}$) is the equilibrium concentration

of I_2 in solution. The Langmuir constant K_L reflects the affinity of the adsorbate for the adsorption sites, while the Freundlich constant K_F represents adsorption capacity related to adsorbate-adsorbent interactions. Finally, 1/n indicates the adsorption intensity, reflecting the energy distribution and heterogeneity of the adsorption sites.[1]

Characterization

Transmission electron microscope (TEM) imagines, high-angle annular dark-field scanning TEM (HAADF-STEM), and elementals mapping analysis were conducted on FEI Tecnai G2-20 transmission electron microscope equipped with energy-dispersive X-ray (EDX). The morphology and shape of materials were characterized by scanning electron microscopy (FE-SEM, inspect F50) with energy-dispersive X-ray (EDX). N_2 adsorption-desorption isotherms were measured with a Belsorp-mini instrument II-Japan at 77 K. Before the adsorption measurements, samples were pretreated at 120 ℃ overnight under vacuum. The surface areas of materials were calculated according to the Brunauer-Emmett-Teller (BET) model by using the adsorption branch data in the relative pressure (P/P_0) range of 0-1.0. The total pore volumes and pore-size distributions were calculated from the adsorption branches of the N_2 adsorption-desorption isotherms by BJH and MP methods. Thermogravimetric (TGA) analysis was performed on a TGA Q50 instrument under air, heated from 50 ℃ to 700 ℃ at a heating rate of 10 °C min^{-1}. Fourier transform infrared (FT-IR) spectra were acquired on a Nicolet IS10 spectrometer (Thermo Scientific) at wavenumber 600-4000 cm^{-1}. Nuclear magnetic resonance spectroscopy (^{1}H-NMR) and ^{13}C-NMR spectra were measured using $CDCl_3$ and H_2SO_4 diluted with DMSO-d6 (1/9, v/v) on a Bruker Advance III 400 MHz NMR spectrometer at 25 °C. CO_2 adsorption isotherms were measured using a BELsorp (II)-mini (MicrotracBEL, Japan) in the pressure range from 0 to 1 bar at 298 K and 323 K. Before adsorption measurement, samples were pretreated at 120 ℃ for 12 h under vacuum to remove any impurities components. X-ray photoelectron spectroscopy (XPS) measurements were carried out using a PHI 5000 VersaProbe (Ulvac-PHI, Japan) equipped with a monochromatic Al K_α (1486.6 eV) X-ray source. Raman spectra were collected with a Renishaw Invia Raman microscope utilizing a 532-nm laser. The iodine concentrations in solutions were measured using UV-vis spectrophotometry (Jasco V-670) within the wavelength range of 200 to 800 nm. X-ray diffraction (XRD)

patterns were performed on the Rigaku diffractometer (Rigaku Smart Lab, Rigaku Co., Japan) (40 kV, 40 mA) using diffracted beam monochromator CuK_α radiation (λ= 1.5406 Å) at a scanning rate of 3° min^{-1} with range 5-40° at 0.01 intervals.

The average crystal size of ZIF-8 were calculated from X-ray spectra using the Debye-Scherrer equation as follows:[5]

$$D_{XRD} = \frac{K\lambda}{(B\ cos\theta)} = \frac{0.9\ \lambda}{(B\ cos\theta)}$$

Where, D_{XRD} is average crystallite size (nm), K is Scherrer constant=0.9, λ is X-ray wavelength of CuK_α radiation (λ= 1.54178 Å), B is the Full Width at Half Maximum (FWHM) of XRD peak, and θ is the Bragg angle of diffraction peak.

The crystallinity in the XRD data was determined by calculating the ratio of the total area beneath the diffraction peaks at 2θ angles of 7.4°, 10.4°, 12.7°, and 18.0° relative to the peak areas in the reference sample, which exhibited a notably crystalline product.[6]

$$Crystallinity = \frac{(\Sigma A)}{(\Sigma A_s)} \times 100$$

Result and discussions

Figures

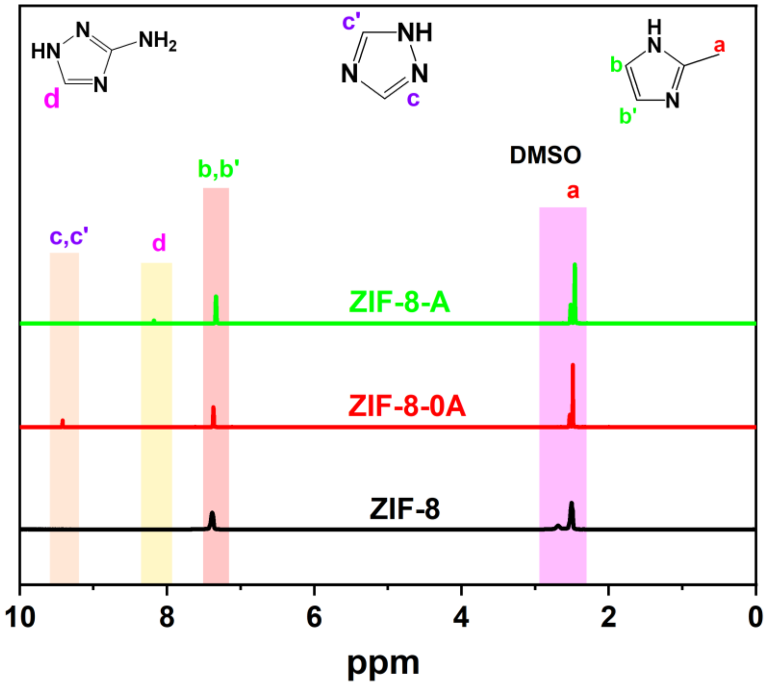

Figure S1. ^{1}H-NMR analysis (DMSO/H_2SO_4=9/1, v/v) of ZIF-8, ZIF-8-0A and ZIF-8-A materials.

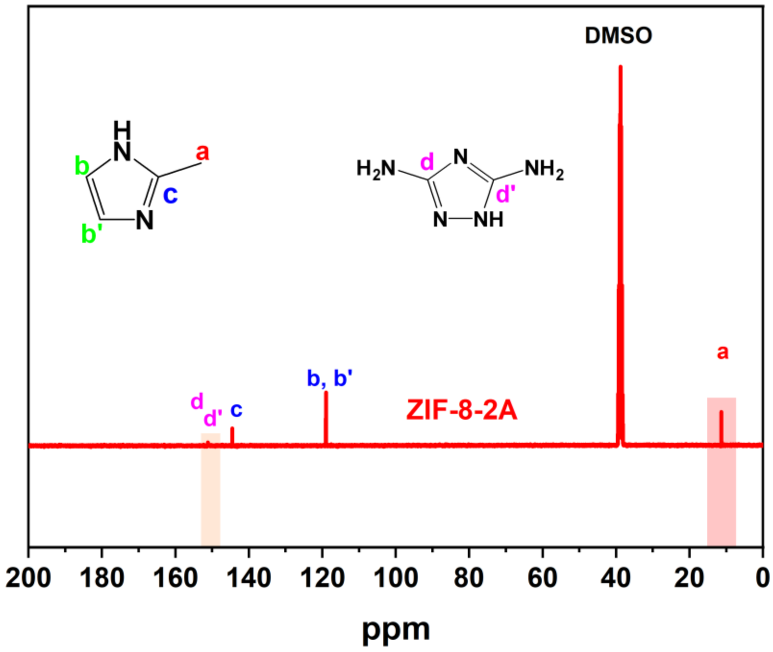

Figure S2. ^{13}C-NMR analysis (DMSO/H_2SO_4=9/1, v/v) of ZIF-8-2A material.

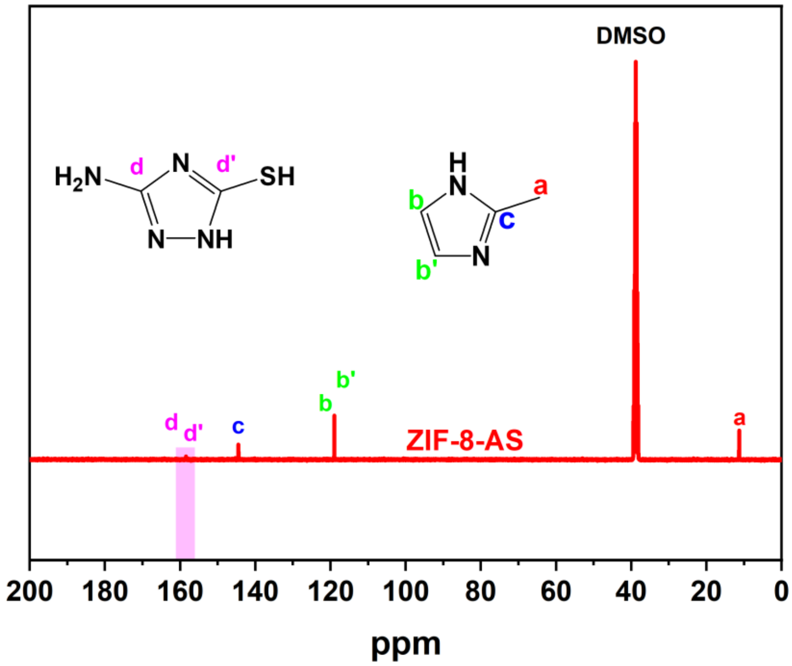

Figure S3. ^{13}C-NMR analysis (DMSO/H_2SO_4=9/1, v/v) of ZIF-8-AS material.

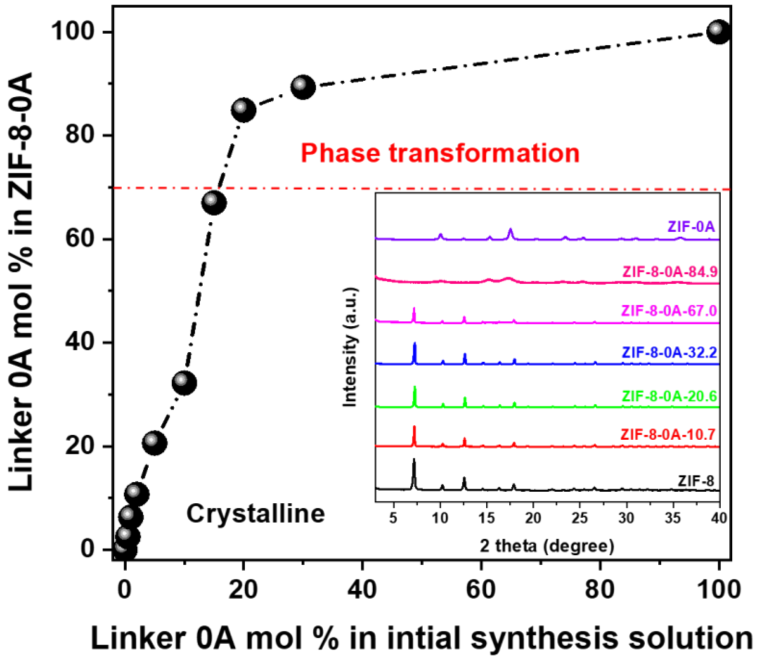

Figure S4. The plot for linker 0A contents in initial synthesis solution vs. linker 0A contents in ZIF-8-0A materials corresponding to XRD patterns.

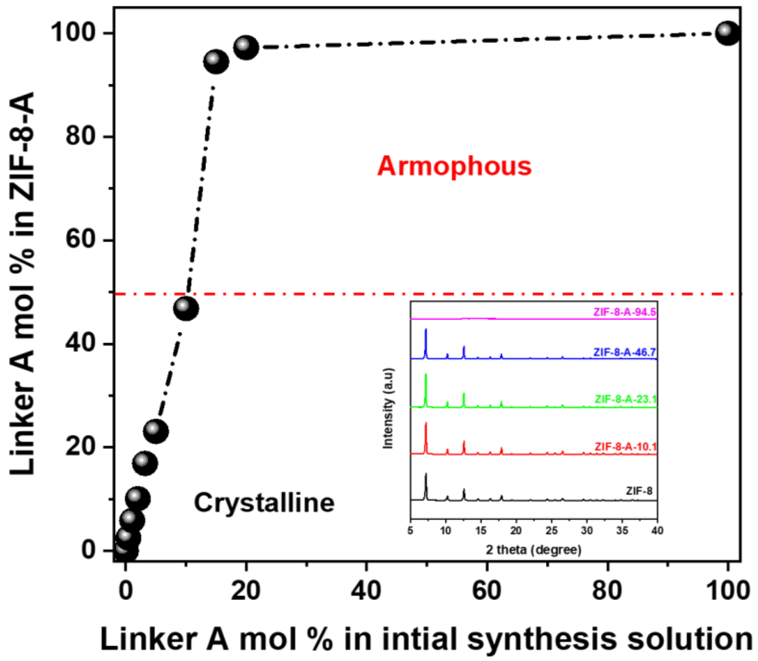

Figure S5. The plot for linker A contents in the initial synthesis solution vs. linker A contents in ZIF-8-A materials corresponding to XRD patterns.

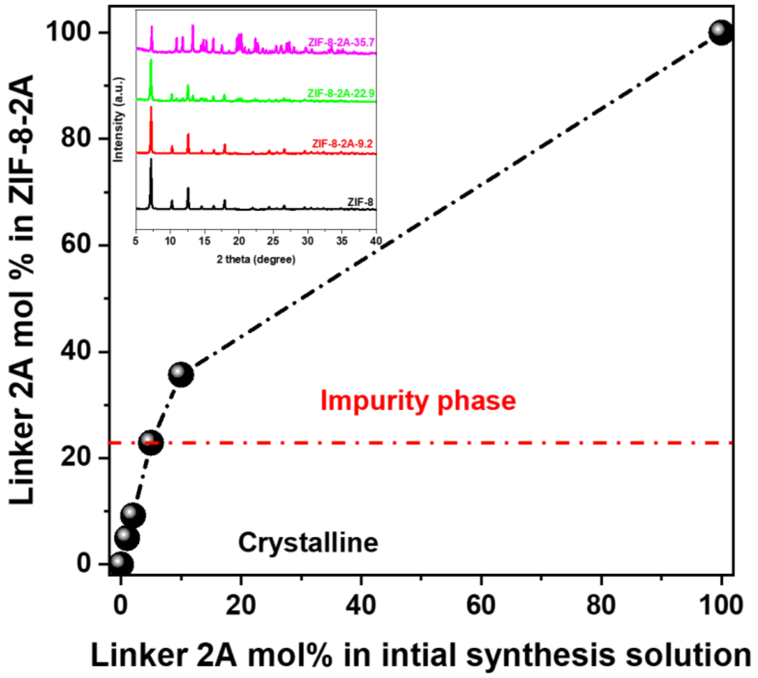

Figure S6. The plot for linker 2A contents in the initial synthesis solution vs. linker 2A contents in ZIF-8-2A materials corresponding to XRD patterns.

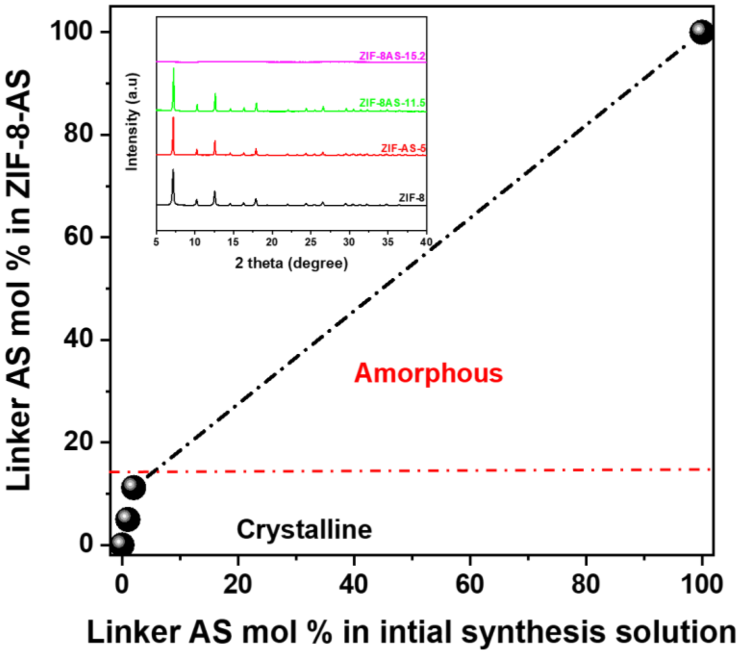

Figure S7. The plot for linker AS content in the initial synthesis solution vs. linker AS contents in ZIF-8-AS materials corresponding to XRD patterns.

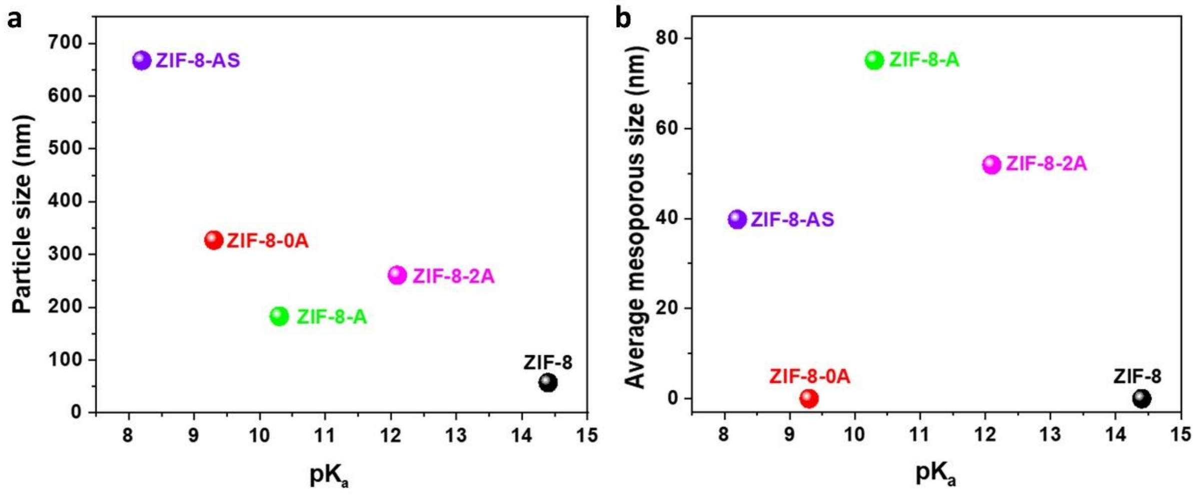

Figure S8. The relationship between (a) particle size (calculated from SEM images) and pK_a value of Hmim in ZIF-8 and linker II (0A, A, 2A, and AS) in ZIF-8-II (ZIF-8-0A, ZIF-8-A, ZIF-8-2A, and ZIF-8-AS) materials and (b) average mesoporous size and pK_a value of linker II in ZIF-8 and functional & hierarchical mesoporous ZIF-8-II materials.

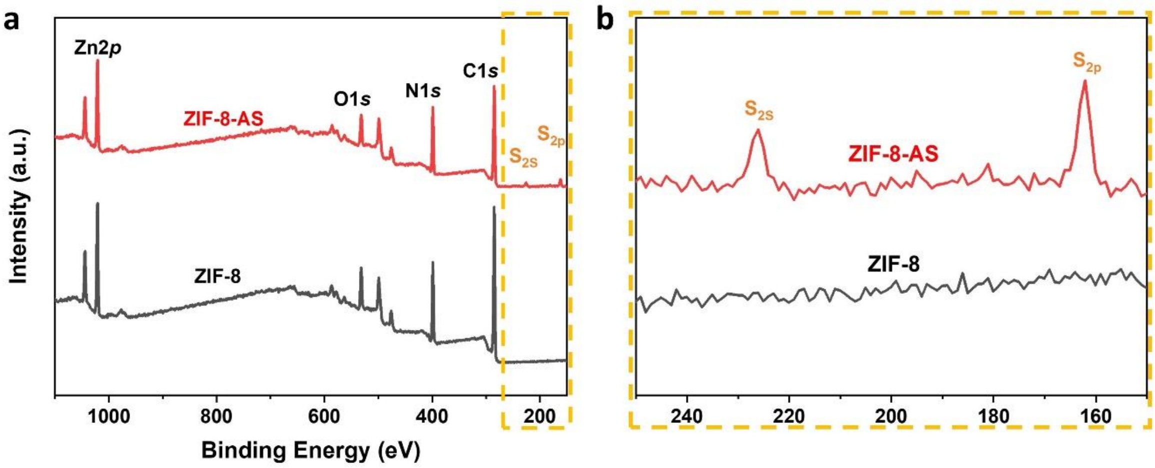

Figure S9. (a) XPS survey spectra with (b) magnified XPS survey spectra in the range of 150-250 eV of ZIF-8 and ZIF-8-AS material.

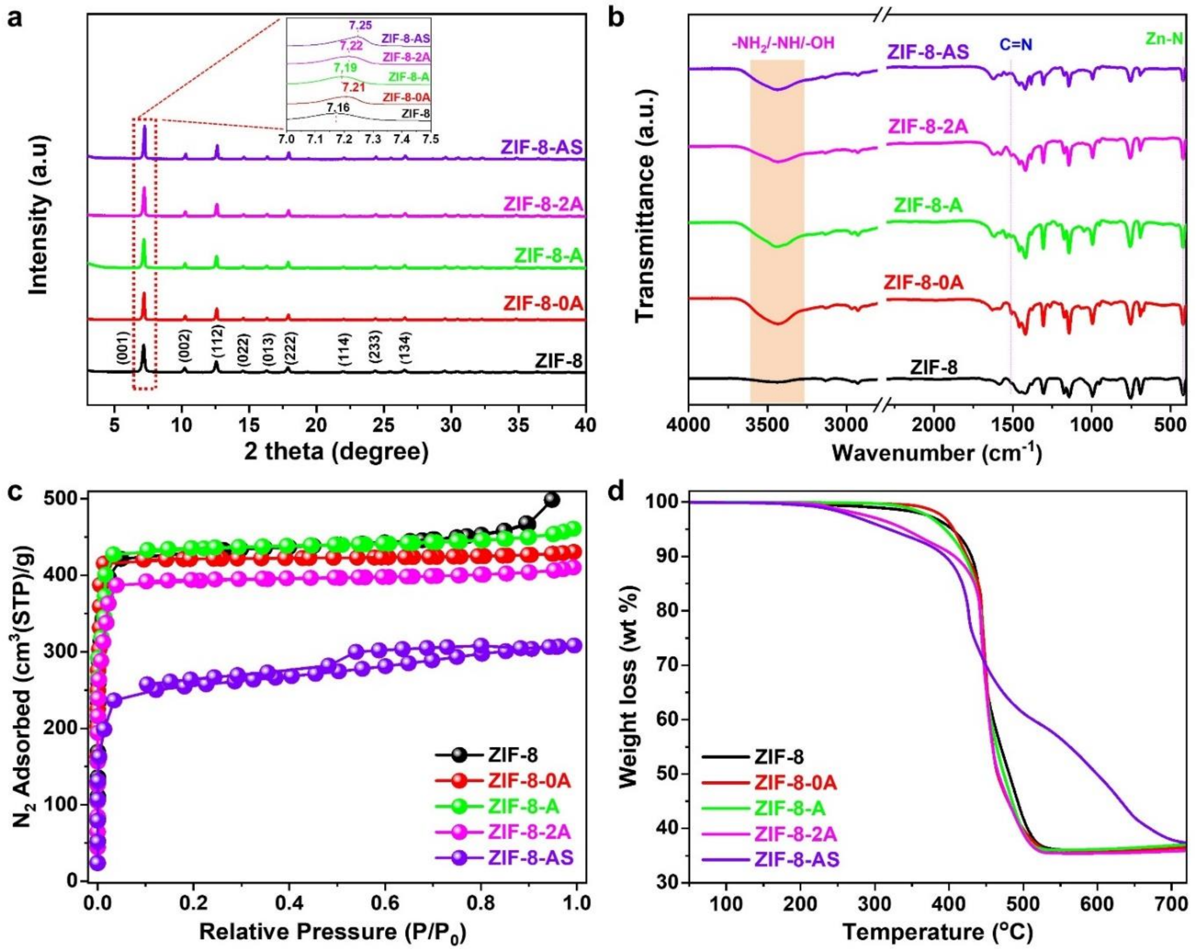

Figure S10. (a) XRD patterns, (b) FT-IR spectra, (c) N_2 adsorption-desorption isotherms, and (d) TGA curves of ZIF-8 and ZIF-8-II.

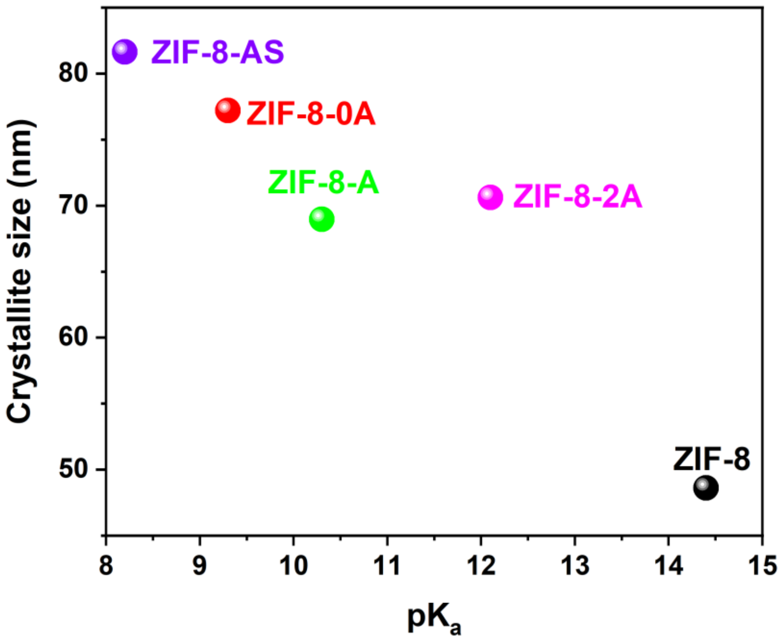

Figure S11. The relationship between crystallite size (calculated from XRD patterns) and pK$_a$ value of Hmim and linker II (0A, A, 2A, and AS) in a functional & hierarchical mesoporous ZIF-8-II materials.

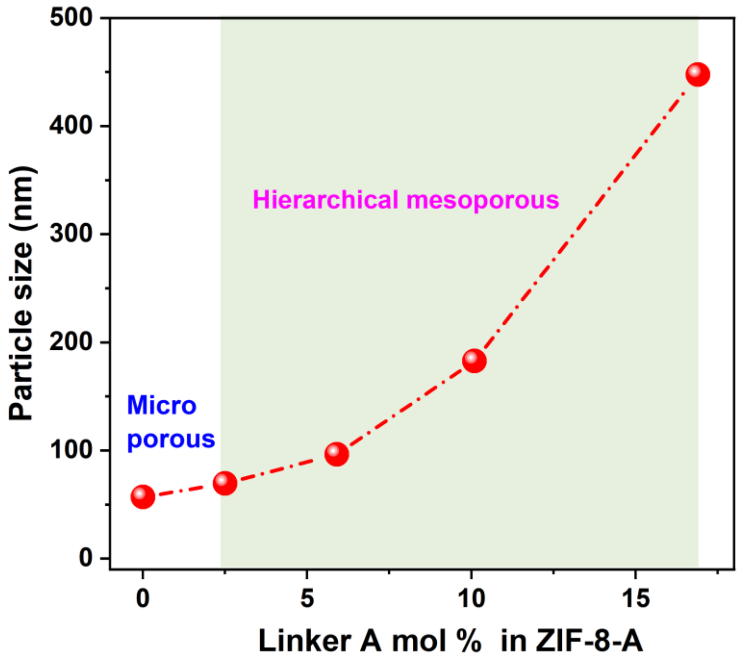

Figure S12. The relationship between particle size of ZIF-8 and amine functional & hierarchical mesoporous ZIF-8-A (calculated from SEM images) and amount of linker A in ZIF-8-A framework.

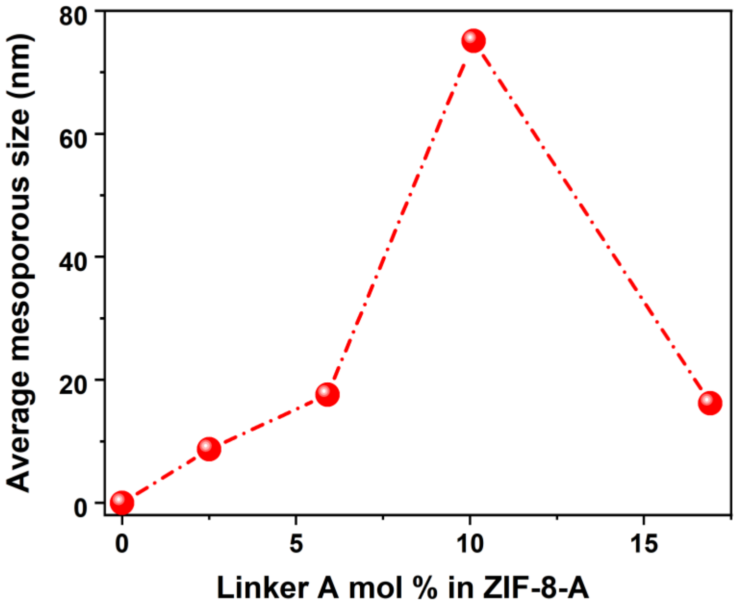

Figure S13. The relationship between average mesoporous size of amine functional & hierarchical mesoporous ZIF-8-A (calculated from TEM images) and amount of linker A in ZIF-8-A materials.

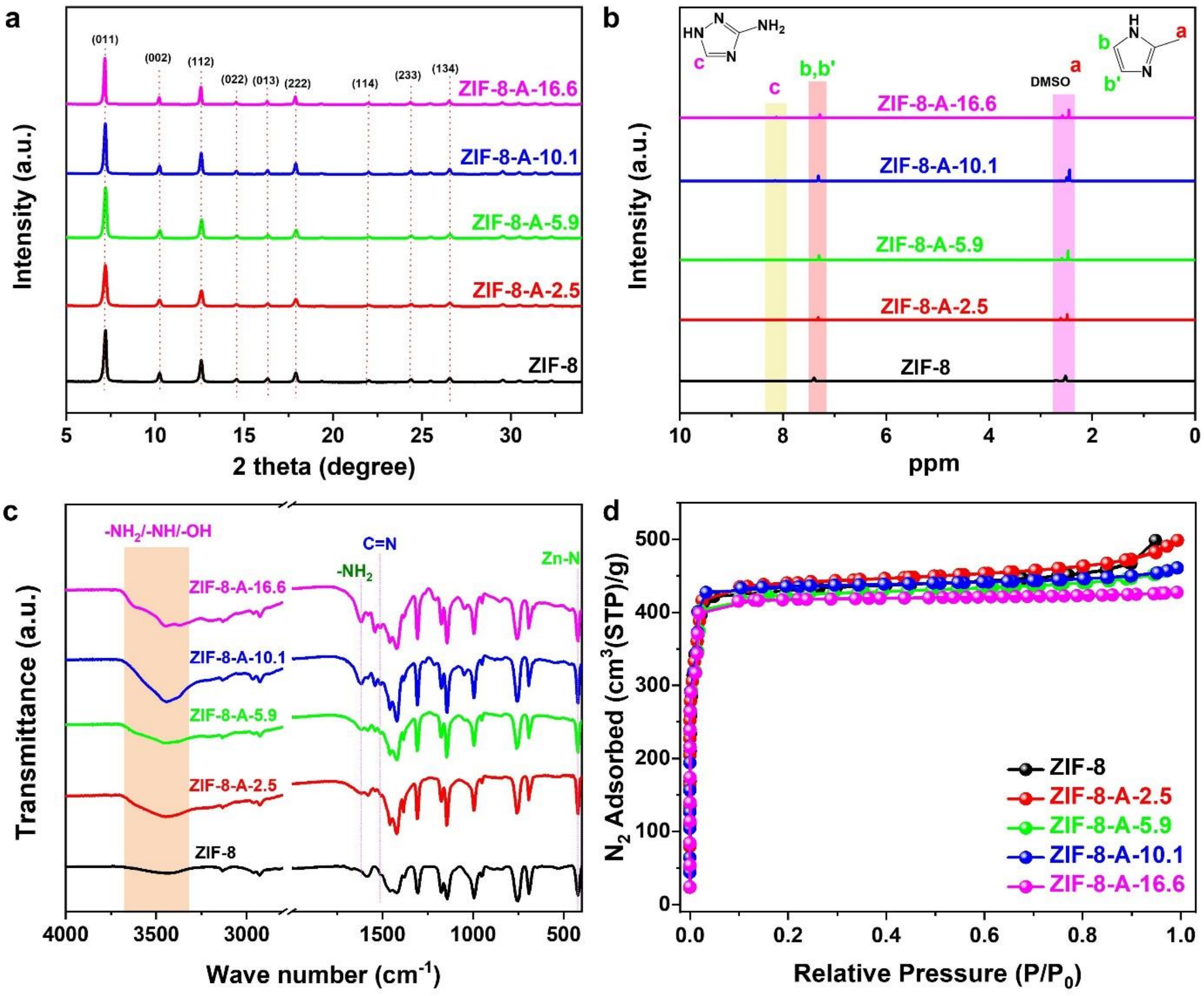

Figure S14. (a) PXRD patterns, (b) ^{1}H-NMR analysis (DMSO/H_2SO_4=9/1, v/v), (c) FT-IR, and (d) N_2 adsorption-desorption isotherms of microporous ZIF-8 and amine functional & hierarchical mesoporous of ZIF-8-A materials.

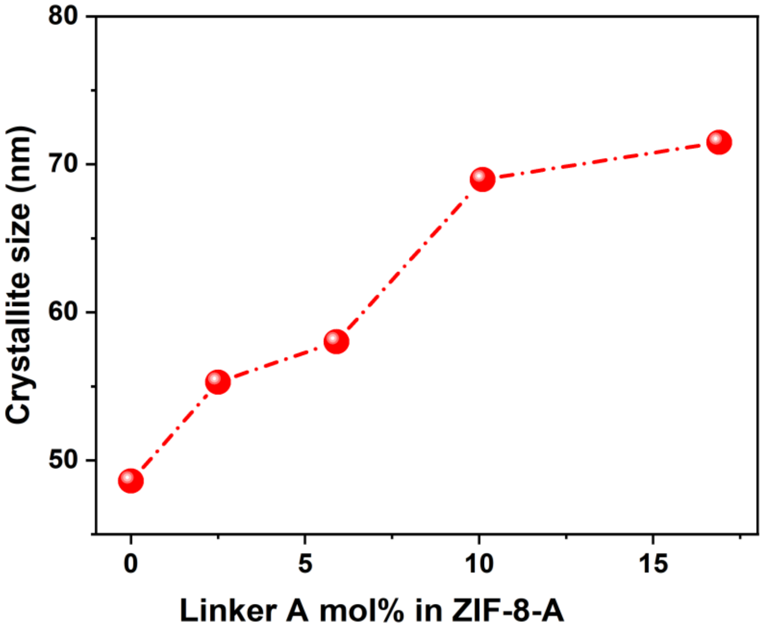

Figure S15. The relationship between crystallite size (calculated from XRD patterns) and amount of linker A in functional & hierarchical mesoporous of ZIF-8-A material.

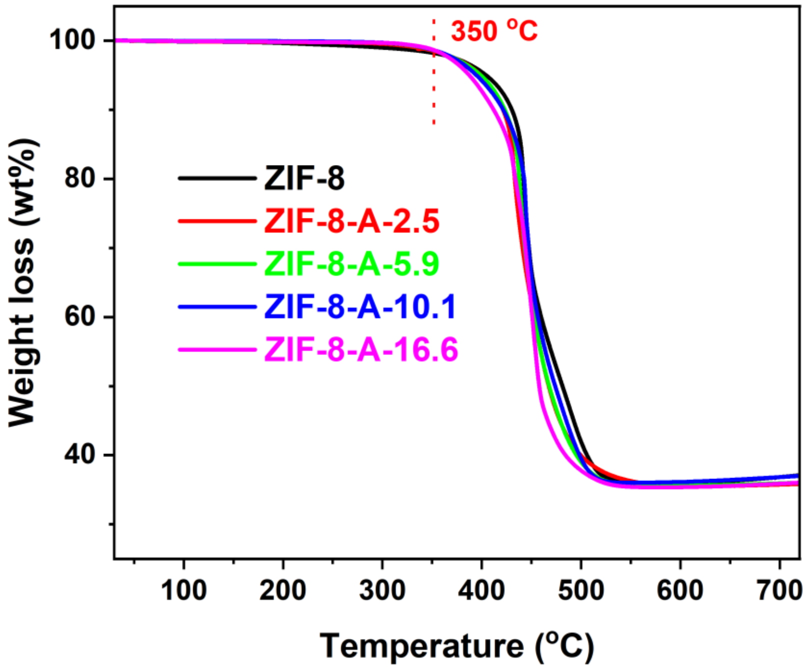

Figure S16. Thermogravimetric analysis (TGA) of ZIF-8 and amine functional & hierarchical mesoporous ZIF-8-A materials in air condition.

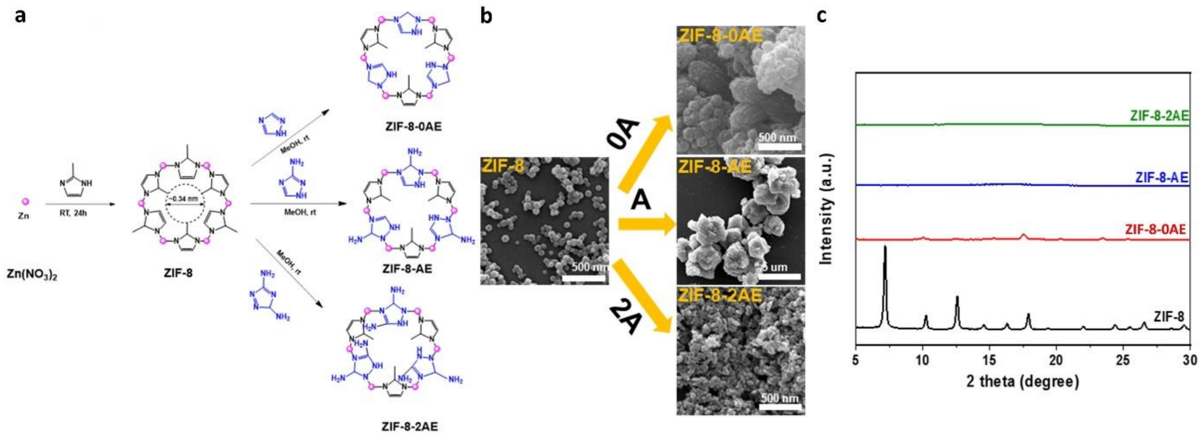

Figure S17. (a) Schematic illustration of post synthetic ligand exchange of ZIF-8 by different ligands (0A, A, and 2A) for 24 hours at room temperature (b) SEM imagines, and (c) XRD patterns of ZIF-8, ZIF-8-0AE, ZIF-8-AE, and ZIF-8-2AE materials.

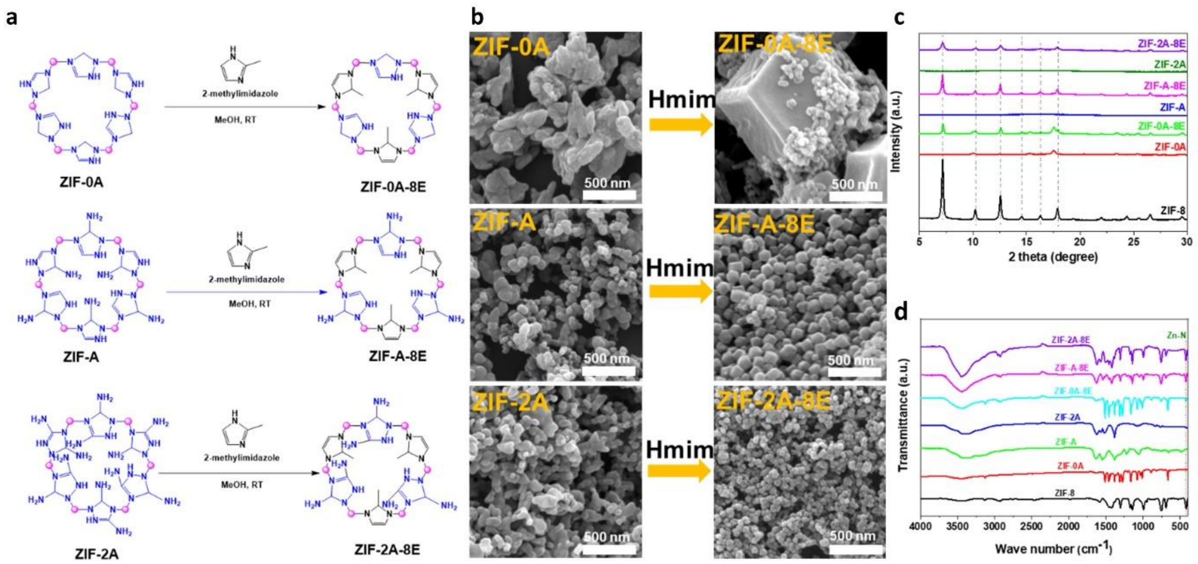

Figure S18. (a) Schematic illustration of post synthetic ligand exchange of ZIF-0A, ZIF-A, ZIF-2A by Hmim ligand for 24 hours at room temperature, (b) SEM imagines, (c) XRD patterns, and (d) FT-IR spectra of ZIF-0A, ZIF-A, ZIF-2A, ZIF-0A-8E, ZIF-A-8E, and ZIF-2A-8E materials.

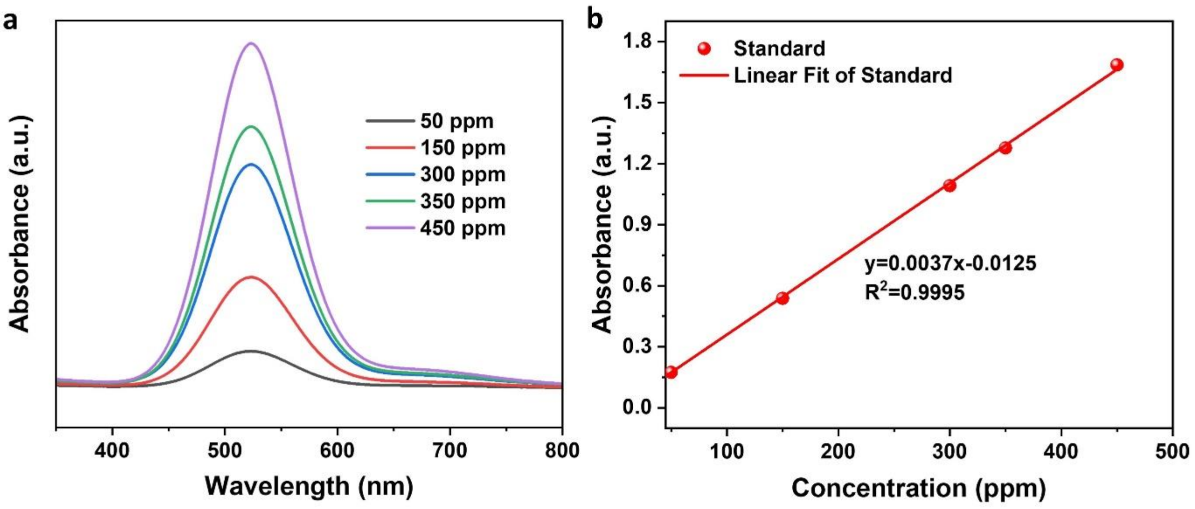

Figure S19. (a) UV-Vis spectra of iodine standard solution in cyclohexane, and (b) The linear curve fit showing the relationship between the concentration and absorbance of iodine standard solution.

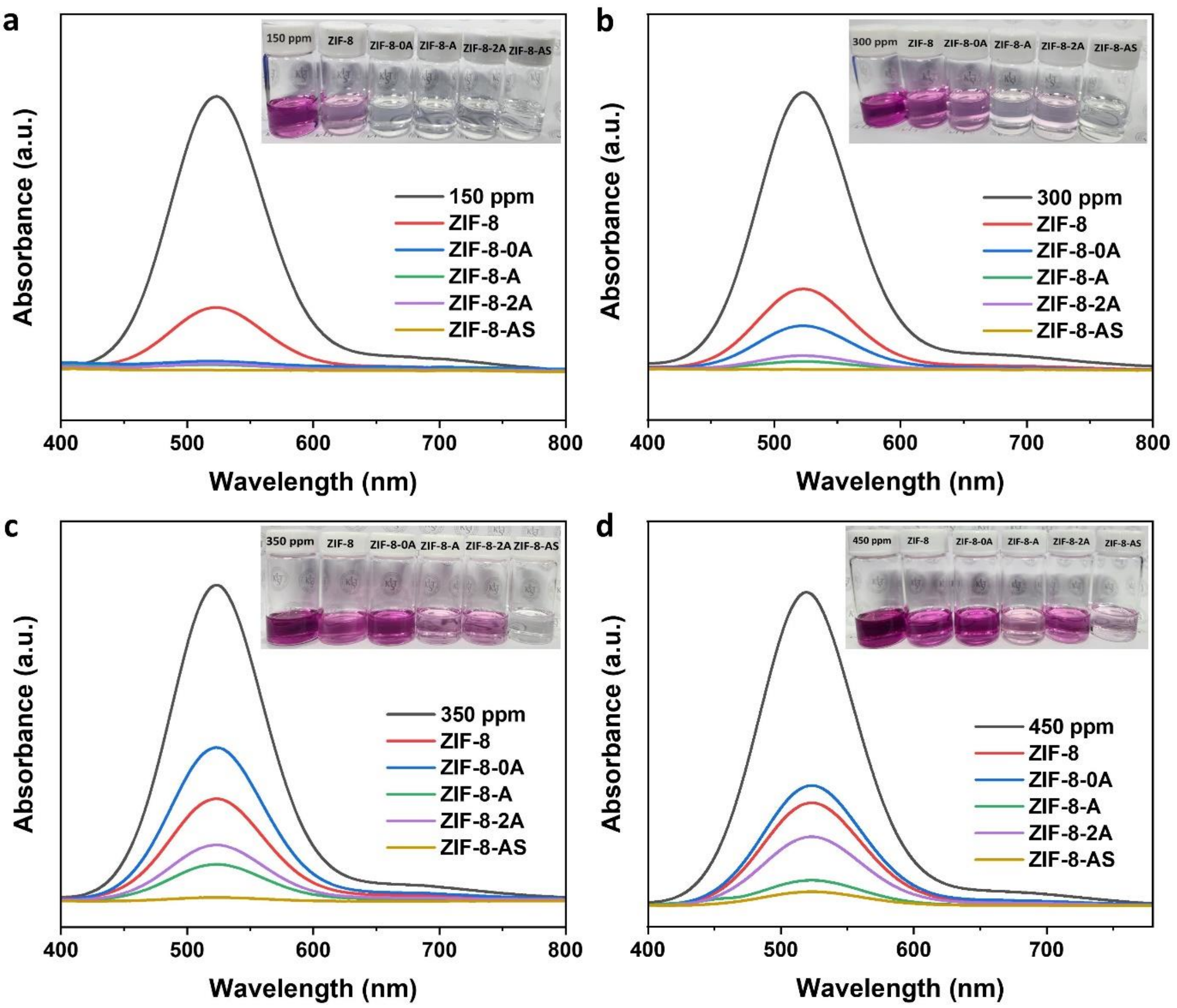

Figure S20. UV-Vis spectra of iodine solution in cyclohexane before and after adsorption at different iodine concentration (in set: color of iodine in cyclohexane before and after 24 h adsorption) (a) 150 ppm, (b) 300 ppm, (c) 350 ppm, and (d) 450 ppm.

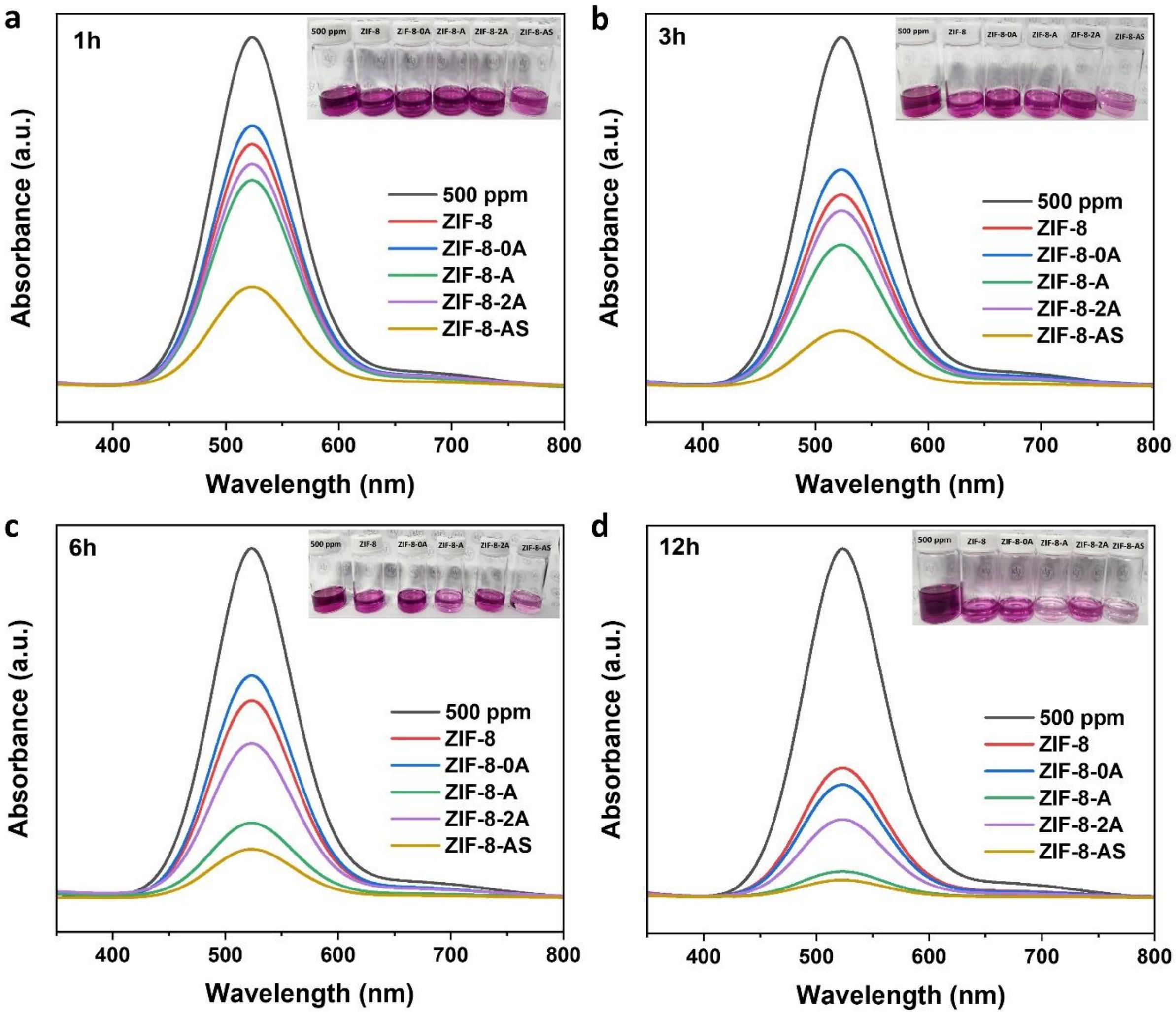

Figure S21. UV-Vis spectra of iodine solution in cyclohexane before and after iodine adsorption with different time adsorption for iodine concentration 500 ppm (in set: color of iodine in cyclohexane before and after adsorption) (a) 1 h, (b) 3 h, (c) 6 h, and (d) 12 h.

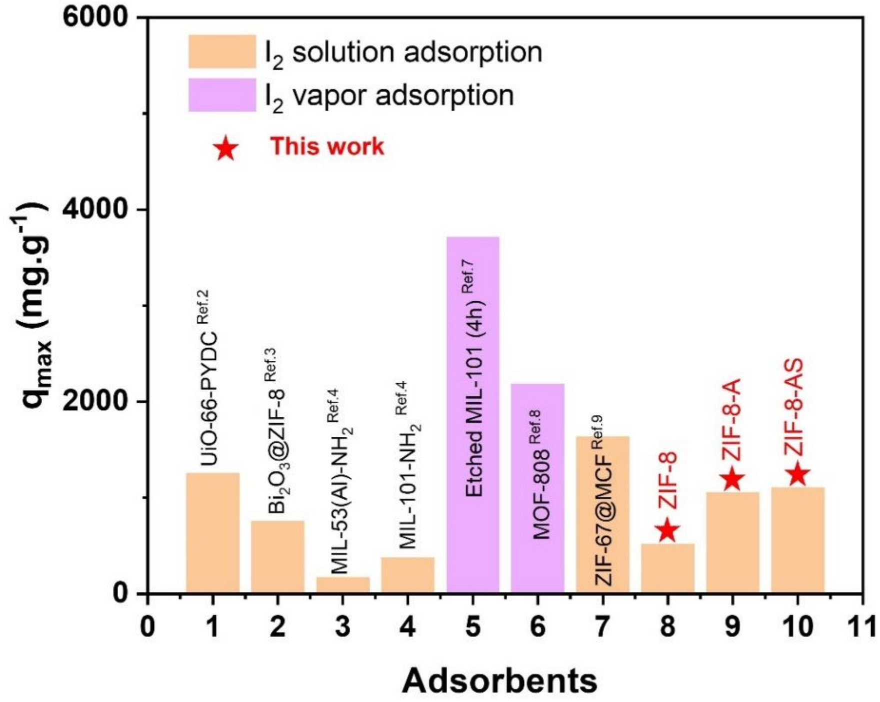

Figure S22. Comparison of iodine adsorption capacities of various adsorbents.

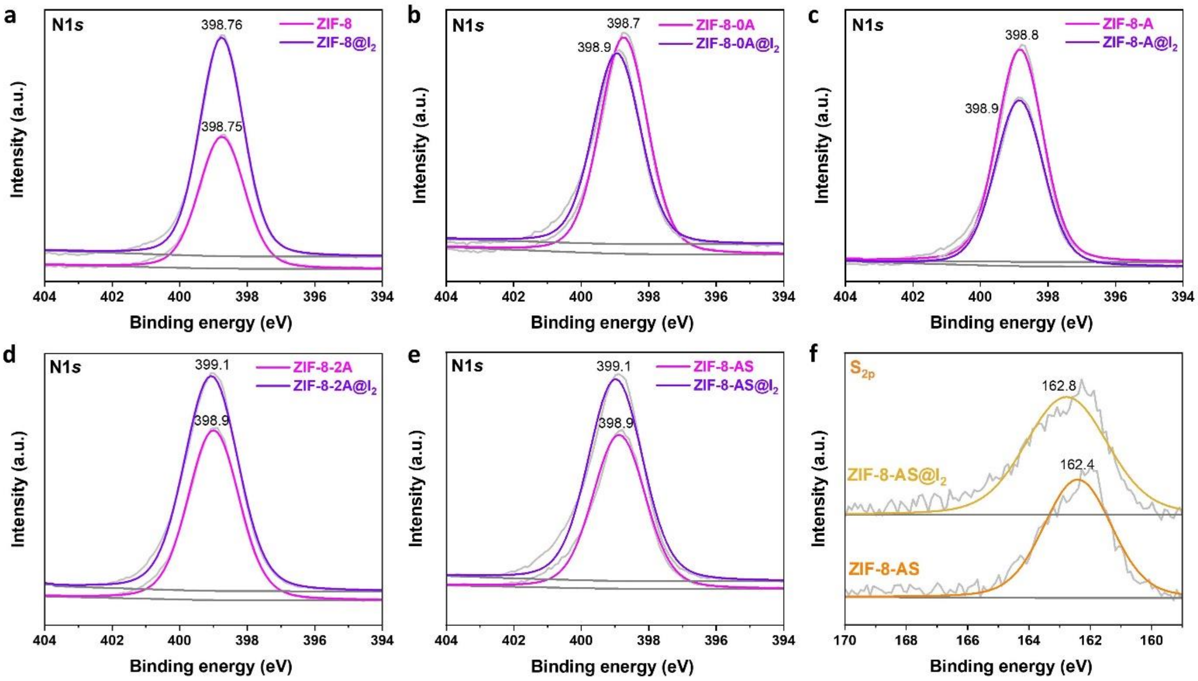

Figure S23. Narrow XPS spectra of ZIF-8 and ZIF-8-II material before and after iodine adsorption. N narrow spectra of (a) ZIF-8, (b) ZIF-8-0A, (c) ZIF-8-A, (d) ZIF-8-2A, (e) ZIF-8-AS, and (f) S narrow spectra of ZIF-8-AS.

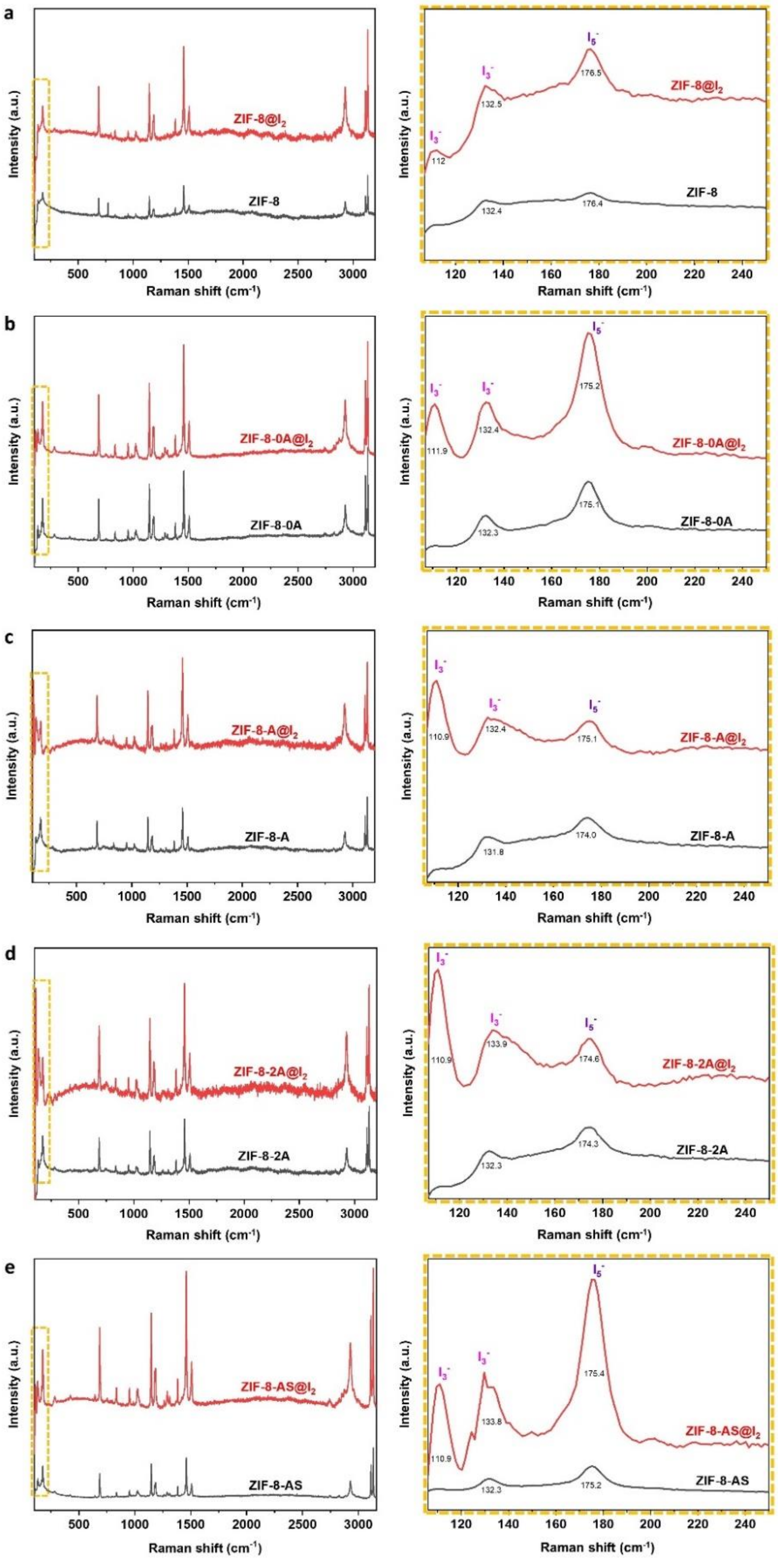

Figure S24. Raman spectra of materials before and after iodine adsorption, with magnified Raman spectra in the range of 100-250 cm^{-1} for (a) ZIF-8, (b) ZIF-8-0A, (c) ZIF-8-A, (d) ZIF-8-2A, and (e) ZIF-8-AS.

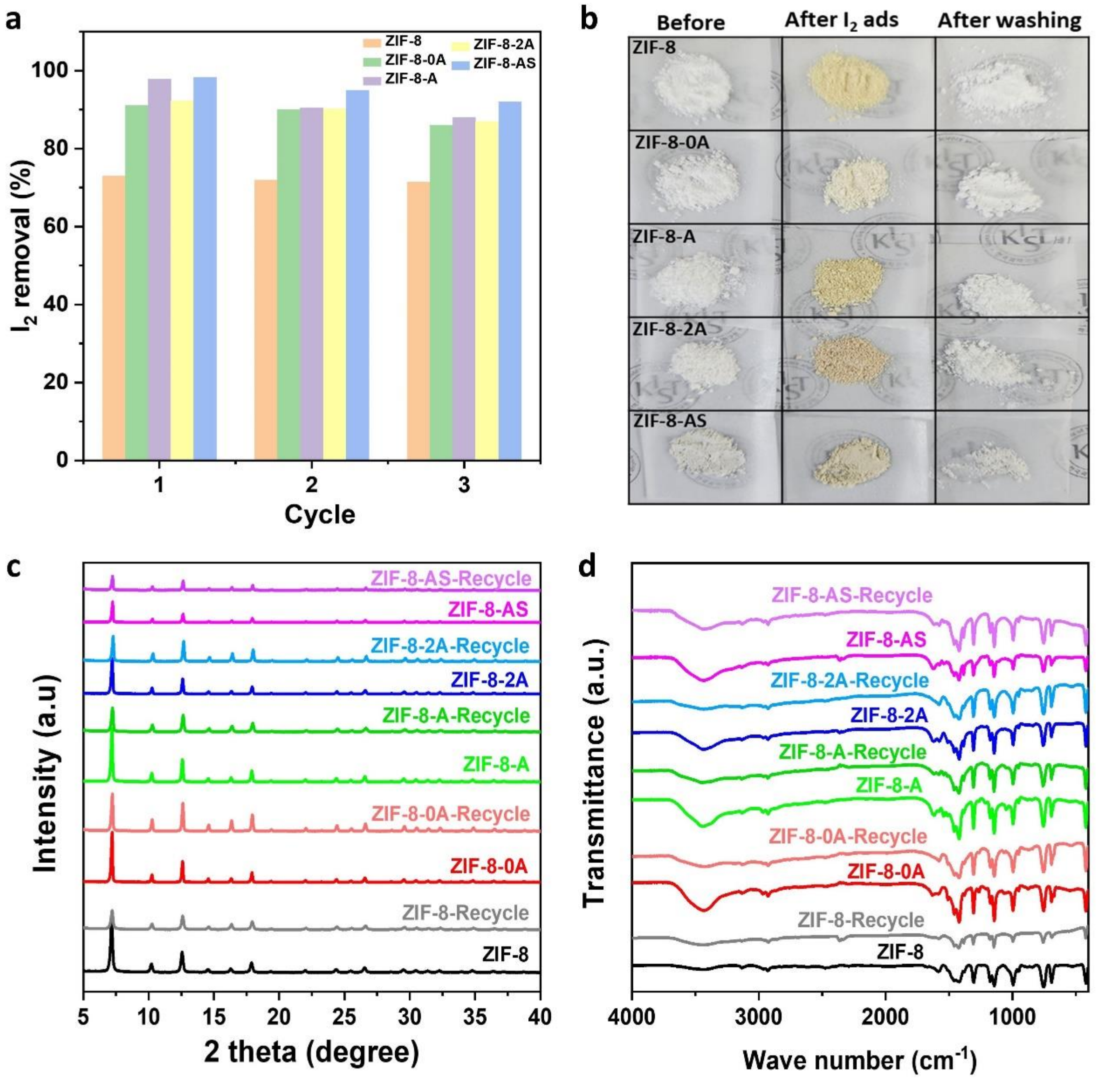

Figure S25. Iodine recyclability and structure characterization of spent materials. (a) The iodine adsorption performance after 3 cycles, (b) photographs of materials before, after I_2 adsorption and after washing by ethanol, (c) XRD patterns of fresh and reused materials, and (d) FT-IR spectra of fresh and reused materials.

Tables

Table S1. Amount of chemical reagent used to synthesize microporous ZIF-8 and multi-functional & hierarchical mesoporous ZIF-8 materials.

Material	Metal source	Ligand		Solvent
	$Zn(NO_3)_2.6H_2O$	Hmim	Linker II	MeOH
ZIF-8	4.76 g, 16 mmol	10.51 g, 128 mmol	NA	480 mL
ZIF-8-0A		10.30g, 125.44 mmol	0.177g, 2.56 mmol 0A	480 mL
ZIF-8-A		10.30g, 125.44 mmol	0.215g, 2.56 mmol A	480 mL
ZIF-8-2A		10.30g, 125.44 mmol	0.254g, 2.56 mmol 2A	480 mL
ZIF-8-AS		10.30g, 125.44 mmol	0.297g, 2.56 mmol AS	480 mL

Table S2. Amount of chemical reagent used to synthesize amine functional & hierarchical mesoporous ZIF-8-A materials.

Material	Metal source	Ligand		Solvent
	$Zn(NO_3)_2{\cdot}6H_2O$	Hmim	Linker A	MeOH
ZIF-8-A-2.5	4.76 g, 16 mmol	10.46 g, 127.45 mmol	0.046 g, 0.55 mmol	480 mL
ZIF-8-A-5.9		10.40 g, 126.63 mmol	0.115 g, 1.37 mmol	480 mL
ZIF-8-A-10.1		10.30 g, 125.44 mmol	0.215 g, 2.56 mmol	480 mL
ZIF-8-A-16.6		10.17 g, 123.90 mmol	0.344 g, 4.10 mmol	480 mL

Table S3. Elemental analysis from XPS analysis for ZIF-8 and ZIF-8-AS materials.

Material	C (% atomic)	N (% atomic)	O (% atomic)	Zn (% atomic)	S (% atomic)
ZIF-8	66.3	18.9	9.4	5.4	0
ZIF-8-AS	59.2	23.8	8.7	6.1	2.22

Table S4. CO_2 adsorption isotherm of ZIF-8 and series of amine functional & hierarchical mesoporous ZIF-8-A materials.

Material	CO_2 uptake (mmol·g^{-1})	
	25 ℃	50 ℃
	1 bar	1 bar
ZIF-8	0.76	0.48
ZIF-8-A-2.5	0.78	0.48
ZIF-8-A-5.9	0.81	0.50
ZIF-8-A-10.1	1.0	0.60
ZIF-8-A-16.6	0.92	0.55

Table S5. Kinetic parameters of the pseudo-first-order and pseudo-second-order models for 100 ppm I_2 adsorption on ZIF-8 and ZIF-8-II materials.

Material	$q_{e,exp}$ (mg·g^{-1})	Pseudo-first-order model			Pseudo-second-order model		
		$q_{e,cal}$ (mg·mg^{-1})	k_1 (h^{-1})	R^2	$q_{e,cal}$ (mg·mg^{-1})	k_2 (g·mg^{-1}·h^{-1})	R^2
ZIF-8	38.3	31.5	1.337	0.813	34.9	4.4×10^{-2}	0.899
ZIF-8-0A	48.3	48	0.164	0.953	57.0	0.25×10^{-2}	0.949
ZIF-8-A	49.6	45.5	1.858	0.931	48.4	5.82×10^{-2}	0.975
ZIF-8-2A	50.2	45.7	1.130	0.937	49.8	3.05×10^{-2}	0.984
ZIF-8-AS	50.0	50	75.320	1	50.6	1	0.998

Table S6. Kinetics parameters of the pseudo-first-order and pseudo-second-order models for 200 ppm I_2 adsorption on ZIF-8 and ZIF-8-II materials.

Material	$q_{e,exp}$ (mg·g^{-1})	Pseudo-first-order model			Pseudo-second-order model		
		$q_{e,cal}$ (mg·mg^{-1})	k_1 (h^{-1})	R^2	$q_{e,cal}$ (mg·mg^{-1})	k_2 (g·mg^{-1}·h^{-1})	R^2
ZIF-8	66.5	61	0.219	0.852	69.6	4.07×10^{-3}	0.900
ZIF-8-0A	85.4	77	0.128	0.978	101.6	1.11×10^{-3}	0.984
ZIF-8-A	95.3	93	0.215	0.929	108.9	2.32×10^{-3}	0.955
ZIF-8-2A	85.4	80	0.177	0.867	94.9	2.15×10^{-3}	0.901
ZIF-8-AS	98.9	95	1.119	0.974	102.2	18×10^{-3}	0.996

Table S7. Kinetics parameters for 500 ppm I_2 adsorption on ZIF-8 and ZIF-8-II materials.

Material	$q_{e,exp}$ (mg·g^{-1})	Pseudo-first-order model			Pseudo-second-order model		
		$q_{e,cal}$ (mg·mg^{-1})	k_1 (h^{-1})	R^2	$q_{e,cal}$ (mg·mg^{-1})	k_2 (g·mg^{-1}·h^{-1})	R^2
ZIF-8	148.0	139	0.361	0.902	155.8	3.27×10^{-3}	0.953
ZIF-8-0A	192.2	204	0.114	0.929	216.8	0.39×10^{-3}	0.936
ZIF-8-A	240.0	233	0.323	0.972	266.4	1.6×10^{-3}	0.979
ZIF-8-2A	203.9	194	0.258	0.919	222	1.54×10^{-3}	0.957
ZIF-8-AS	241.3	224	1.409	0.973	237.8	10.1×10^{-3}	0.993

Table S8. The relationship between BET surface area, poresize, functional groups and iodine adsorption capacity.

Material	BET surface ($m^2 \cdot g^{-1}$)	V_{pore} ($cm^3 \cdot g^{-1}$)	Average mesoporous (nm)	Functional groups	Iodine adsorption capacity maximum ($mg \cdot g^{-1}$)
ZIF-8	1758	0.771	0	-2N	514.3
ZIF-8-0A	1738	0.666	0	-3N	862.5
ZIF-8-A	1583	0.712	75.1	-3N,-NH_2	1050.0
ZIF-8-2A	1614	0.634	51.9	-3N,-2NH_2	908.8
ZIF-8-AS	961	0.476	39.8	-3N,-NH_2,-SH	1101.5

Table S9. Comparison of iodine adsorption capacities of various adsorbents.

Material	Solvent	Time	Fitted model	Capacity Max ($mg \cdot g^{-1}$)	Ref.
UiO-66-PYDC	cyclohexane	24	Langmuir	1250	[2]
Bi_2O_3@ZIF-8	cyclohexane	12	Langmuir	751.4	[3]
MIL-53(Al)-NH_2	cyclohexane	48	Langmuir	170	[4]
MIL-101-NH_2	cyclohexane	48	Langmuir	375	[4]
Etched MIL-101 (4h)	Vapor	8	-	3710	[7]
MOF-808	Vapor	24	-	2180	[8]
ZIF-67@MCF	cyclohexane	10	Langmuir	1630	[9]
ZIF-8	Cyclohexane	24	Langmuir	514.3	This work
ZIF-8-A	Cyclohexane	24	Langmuir	1050.0	This work
ZIF-8-AS	Cyclohexane	24	Langmuir	1101.5	This work

References

[1] K. Y. Baek, Y. R. Lee, X. H. Do, K. Y. Cho, K. Jeong, *ACS Appl. Nano Mater.* **2020**, *3*, 9852–9861.

[2] Z. Wang, Y. Huang, J. Yang, Y. Li, Q. Zhuang, J. Gu, *Dalt. Trans.* **2017**, *46*, 7412–7420.

[3] Z. W. Wang, K. W. Chen, A. T. Gu, X. Y. Zhou, P. Wang, C. H. Gong, P. Mao, Y. Jiao, K. Chen, J. G. Lu, Y. Yang, *J. Solid State Chem.* **2023**, *325*, 124186.

[4] C. Falaise, C. Volkringer, J. Facqueur, T. Bousquet, L. Gasnot, T. Loiseau, *ChemComm* **2013**, *49*, 10320–10322.

[5] M. N. Timofeeva, I. A. Lukoyanov, V. N. Panchenko, K. I. Shefer, M. S. Mel'gunov, B. N. Bhadra, S. H. Jhung, *Mol. Catal.* **2022**, *529*, 112530.

[6] A. Akhundzadeh Tezerjani, R. Halladj, S. Askari, *RSC Adv.* **2021**, *11*, 19914–19923.

[7] P. Wang, B. B. Qi, A. T. Gu, K. W. Chen, C. H. Gong, Y. Yi, *Adsorpt. Sci. Technol.* **2023**, 2023.

[8] P. Chen, X. He, M. Pang, X. Dong, S. Zhao, W. Zhang, *ACS Appl. Mater. Interfaces* **2020**, *12*, 20429–20439.

[9] L. Chen, J. Y. Qian, D. D. Zhu, S. Yang, J. Lin, M. Y. He, Z. H. Zhang, Q. Chen, *ACS Appl. Nano Mater.* **2020**, *3*, 5390–5398.
